# Supplementary material for: Discovery, recognized antigenic structures, and evolution of cross-serotype broadly neutralizing antibodies from porcine B-cell repertoires against foot-and-mouth disease virus
Source: PLoS Pathog. 2024 Oct 15;20(10):e1012623. doi: 10.1371/journal.ppat.1012623 (PMC11508087; doi:10.1371/journal.ppat.1012623)
Supplement: S1 Appendix — Fig A. Sorting of FMDV-specific B cells using different bait antigens by fluorescence-activated cell sorting (FACS). (A) Distribution and proportion of FMDV O serotype (O/18074) of specific B cells in the porcine peripheral blood mononuclear cells (PBMCs) identified by flow cytometry. Gate 1 (P1) was selected to exclude cell debris, with lower values of SSC-A and FSC-A, and further analyzed singlets in gate P2 based on diagonal streak of the FSC-A and FSC-H plot. The class-switched (IgG+) B cells in gate P3 were used to check the distribution of FMDV-specific cells, in the presence of FMDV serotype O (O/18074) bait antigen. Appropriately one million counts were collected to analyze the proportion of O/18074-binding B cells in PBMCs. (B) Sorting of the FMDV A serotype (A/AF72) or FMDV O serotype (O/18074) specific B from the enriched B cells population using FACS. After excluding cell debris to gate singlets in P2, the class-switched B cells being IgM-CD14-CD3-CD335- population were gated in P3 to sort the FMDV-specific cells in the presence of bait antigen A/AF72 and O/18074, respectively. (C) Determination of the purity of the sorted FMDV-specific B cells by flow cytometry. After sorting, the purified B cells were reloaded and one thousand counts were collected to check the proportion of O/18074-binding B cells. Fig B. The phenotype and constitution of FMDV-binding cells revealed by scRNA-seq transcripts. (A) UMAP plot of unsupervised clustering of FMDV-binding cells, comprising of majority B cells, as well as other minimal cell populations such as monocytes, T cell, dendritic cells (DCs) and unidentified cells. (B) Heatmap of differentially expressed genes in B cells, Monocytes, T cells, DCc, and others. Fig C. The heterogeneity of porcine memory B cells revealed by pairing analysis of BCR and transcripts. (A) Heatmap of differentially expressed genes between memory B cells and PBs. (B-F) UMAP plot of unsupervised clustering of porcine memory B cells, comprising o [file ppat.1012623.s001.docx]

**Supporting Information for**

**Discovery, recognized antigenic structures, and evolution of cross-serotype broadly neutralizing antibodies from porcine B-cell repertoires against foot-and-mouth disease virus**

Fengjuan Li^1,3#^, Shanquan Wu^2#^, Lv Lv^1,3^, Shulun Huang^1,3^, Zelin Zhang^2,3^, Zhaxi Zerang^2,3^, Pinghua Li^1,3^, Yimei Cao^1,3^, Huifang Bao^1,3^, Pu Sun^1,3^, Xingwen Bai^1,3^, Yong He^4^, Yuanfang Fu^1,3^, Hong Yuan^1,3^, Xueqing Ma^1,3^, Zhixun Zhao^1,3^, Jing Zhang^1,3^, Jian Wang^1,3^, Tao Wang^1,3^, Dong Li^1,3^, Qiang Zhang^1,3^, Jijun He^1,3^, Zaixin Liu^1,3*^, Zengjun Lu^1,3*^, Dongsheng Lei^2,1*^, Kun Li^1,3*^

^1^ State Key Laboratory for Animal Disease Control and Prevention, College of Veterinary Medicine, Lanzhou University, Lanzhou Veterinary Research Institute, Chinese Academy of Agricultural Sciences, Lanzhou, China.

^2^ School of Physical Science and Technology, Electron Microscopy Centre of Lanzhou University, Lanzhou University, Lanzhou, China.

^3^Gansu Province Research Center for Basic Disciplines of Pathogen Biology, Lanzhou, China.

^4^ School of Pharmaceutical Sciences, Shandong University, Ji'nan, Shandong Province, P. R. China.

^#^These authors contributed equally to this work.

*[liuzaixin@caas.cn](mailto:liuzaixin@caas.cn) (ZL) [luzengjun@caas.cn](mailto:luzengjun@caas.cn) (ZeL) [leids@lzu.edu.cn](mailto:leids@lzu.edu.cn) (DL) [likun02@caas.cn](mailto:likun02@caas.cn) (KL).

**This SI file includes:**

Supporting text.

Fig A to N.

Table A to I.

SI References.

**Supporting text**

**Section A:**

**Pairing analysis of BCR and transcriptome reveals the porcine antibodies were derived from plasmablasts and heterogenous memory B cells**

The phenotypes of FMDV-specific B cells were examined using single cell RNA sequencing (scRNA-seq) to elucidate the contribution of different B cell subsets in shaping the porcine antibody repertoire. The uniform manifold approximation and projection (UMAP) clustering analysis of the constructed library revealed that 98% of FMDV-binding cells were identified as porcine B cells, characterized by high expression levels of BCR complex molecules CD79A/B and membrane spanning 4-domains A1 (MS4A1) (Fig B, panel A). In contrast, other minimal cell populations such as monocytes (expressing CD14, FCGR3A and LYZ), T cells (expressing CD3D and CD3E), dendritic cells (DCs) (expressing FCER1A) and unidentified cells appeared as spotted cell groups (Fig B, panel B). Furthermore, the B cells population was pulled and subjected to clustering analysis, revealing the heterogeneity of FMDV-specific B cells. Notably, two distinct subpopulations were observed within the repertoire of FMDV-specific B cells (Fig 1E). The population (cluster 9) was distinctly segregated from other clusters and identified as porcine plasmablasts, based on our observation of significant upregulation of genes (avg_log2FC >2) involved in the joining chain of multimeric IgA and IgM (JCHAIN), protein refolding (TXNDC5, PDIA4, HSP90B1, SDF2L1, FKBP11 and CRELD2), ER-to-Golgi transport (LMAN1, MANF and SSR1), as well as X-box binding protein 1 (XBP1), an evolutionarily conserved transcription factor associated with terminal differentiation of B lymphocytes to plasma cells and production of immunoglobulins (1) (Fig C, panel A). The remaining clusters were consolidated into the other population and identified as porcine memory B cells based on the presence of class switch and SHM accumulation. However, differential gene expression analysis did not reveal any genes specifically representing memory B cells, indicating the heterogeneity of porcine memory B cell populations.

The heterogeneity of memory B cells in mouse and human were intensively dissected at single cell transcriptional level to provide insights into regulation of cells differentiation, BCR diversity and antibody affinity mature(2, 3). Next, we focused on porcine memory B cells and performed pairing analysis of BCR and transcriptome to characterize B cells subsets response to FMDV. We found that the germline V gene usage for light chain of porcine antibody showed obvious bias in the memory B cells population (Fig C, panel B). As depicted in Fig C, panel C-F, light chain V gene usage of distinct subsets of porcine B cells are observed, specifically lambda V8-expressing B cells (comprising 97% of cells in clusters 0, 1 and 5), kappa V1-expressing B cells (constituting 65% of cells in cluster 3), kappa V2-expressing B cells (constituting 50% of cells in cluster 2), and lambda V3-expressing B cells (constituting 42% of cells in cluster 4). This heterogeneity character suggests a potential relationship between BCR recognition and B cell phenotype in porcine memory B cells.

**Section B:**

**The functional antigenic determinants on FMDV revealed by porcine bnAbs-escapes mutants**

To identify crucial functional determinants involving these epitopes on viable viral particle, the neutralization escape mutants were selected for the 10 porcine-derived bnAbs against O/HN/CHA/93 isolate following the same protocol in our previous description(4). As shown in Table C, a novel substitution on VP2 at residue 68 (D) appeared solely in all pOA-2 escape mutants. Contrastively, for the remaining bnAbs escape mutants, the substitutions were primarily focused on VP1 G-H loop close to the RGD motif, the known integrin receptor binding site on FMDV. Concretely, the substitution on VP1 148 position (RGD +1) residue highly occupied in the pOA-6 (7/8), pOA-13 (8/8), pOA-17 (2/3) and pOA-20 (8/8) escape mutants, whereas the substitution on VP1 149 position (RGD +2) residue biased in the pOA-1 (4/7), pOA-7 (5/6), pOA-8 (4/6), pOA-9 (4/5) and pOA-19 (8/8) escape mutants. In addition, accompanying the VP1 148/149 position substitution, several sporadic mutations randomly occurred in a few of neutralization escape mutants with lower frequency and partially similar charged residues substitution, such as VP1 83 (K→R) and 108 (H→R), thus no affection on the antibodies binding with FMDV. The results above indicated three critical residues on the surface of FMDV involving the 10 bnAbs recognition.

**Fig A to N**

**
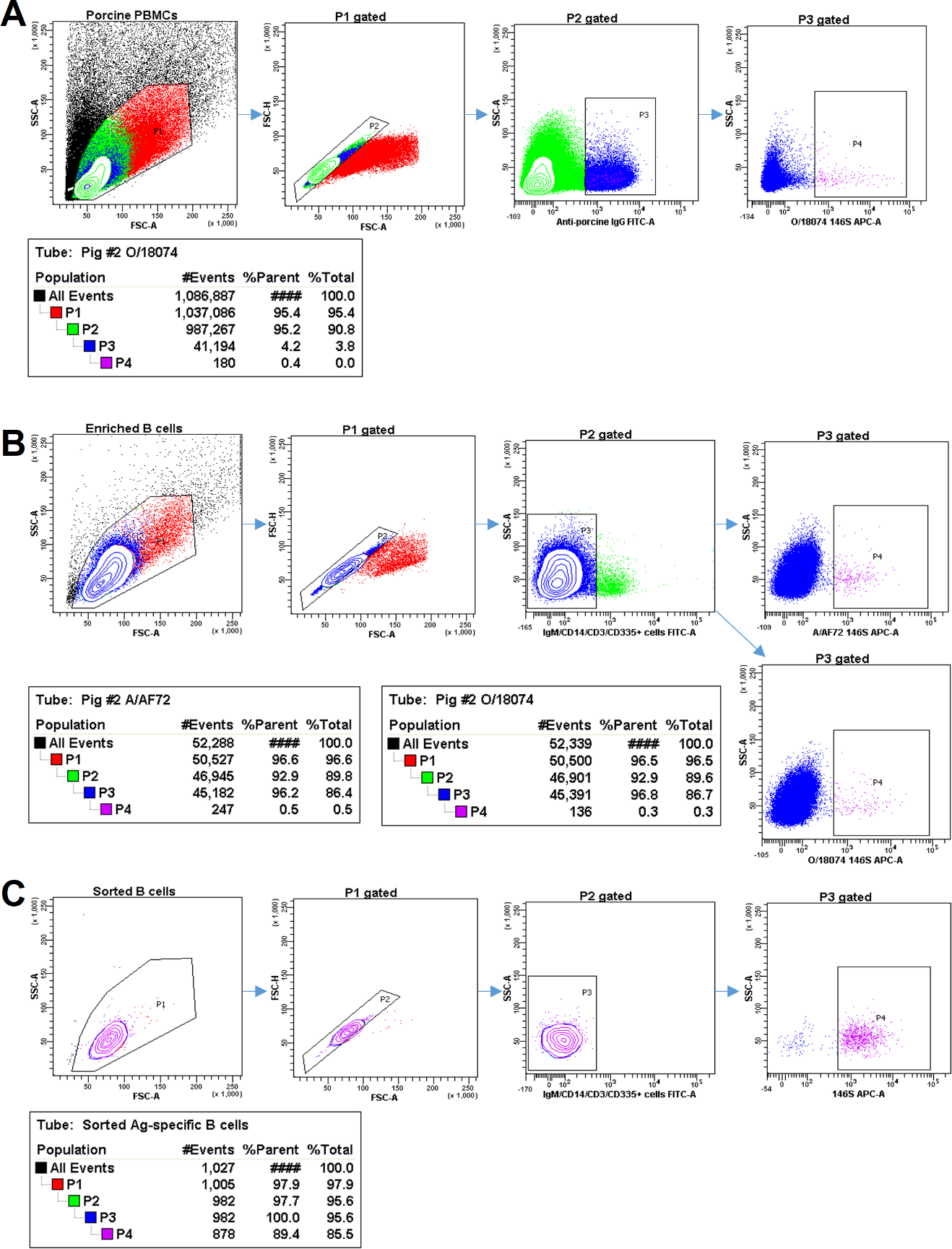
**

**Fig A. Sorting of FMDV-specific B cells using different bait antigens by fluorescence-activated cell sorting (FACS). (A)** Distribution and proportion of FMDV O serotype (O/18074) of specific B cells in the porcine peripheral blood mononuclear cells (PBMCs) identified by flow cytometry. Gate 1 (P1) was selected to exclude cell debris, with lower values of SSC-A and FSC-A, and further analyzed singlets in gate P2 based on diagonal streak of the FSC-A and FSC-H plot. The class-switched (IgG^+^) B cells in gate P3 were used to check the distribution of FMDV-specific cells, in the presence of FMDV serotype O (O/18074) bait antigen. Appropriately one million counts were collected to analyze the proportion of O/18074-binding B cells in PBMCs. **(B)** Sorting of the FMDV A serotype (A/AF72) or FMDV O serotype (O/18074) specific B from the enriched B cells population using FACS. After excluding cell debris to gate singlets in P2, the class-switched B cells being IgM^-^CD14^-^CD3^-^CD335^-^ population were gated in P3 to sort the FMDV-specific cells in the presence of bait antigen A/AF72 and O/18074, respectively. **(C)** Determination of the purity of the sorted FMDV-specific B cells by flow cytometry. After sorting, the purified B cells were reloaded and one thousand counts were collected to check the proportion of O/18074-binding B cells.


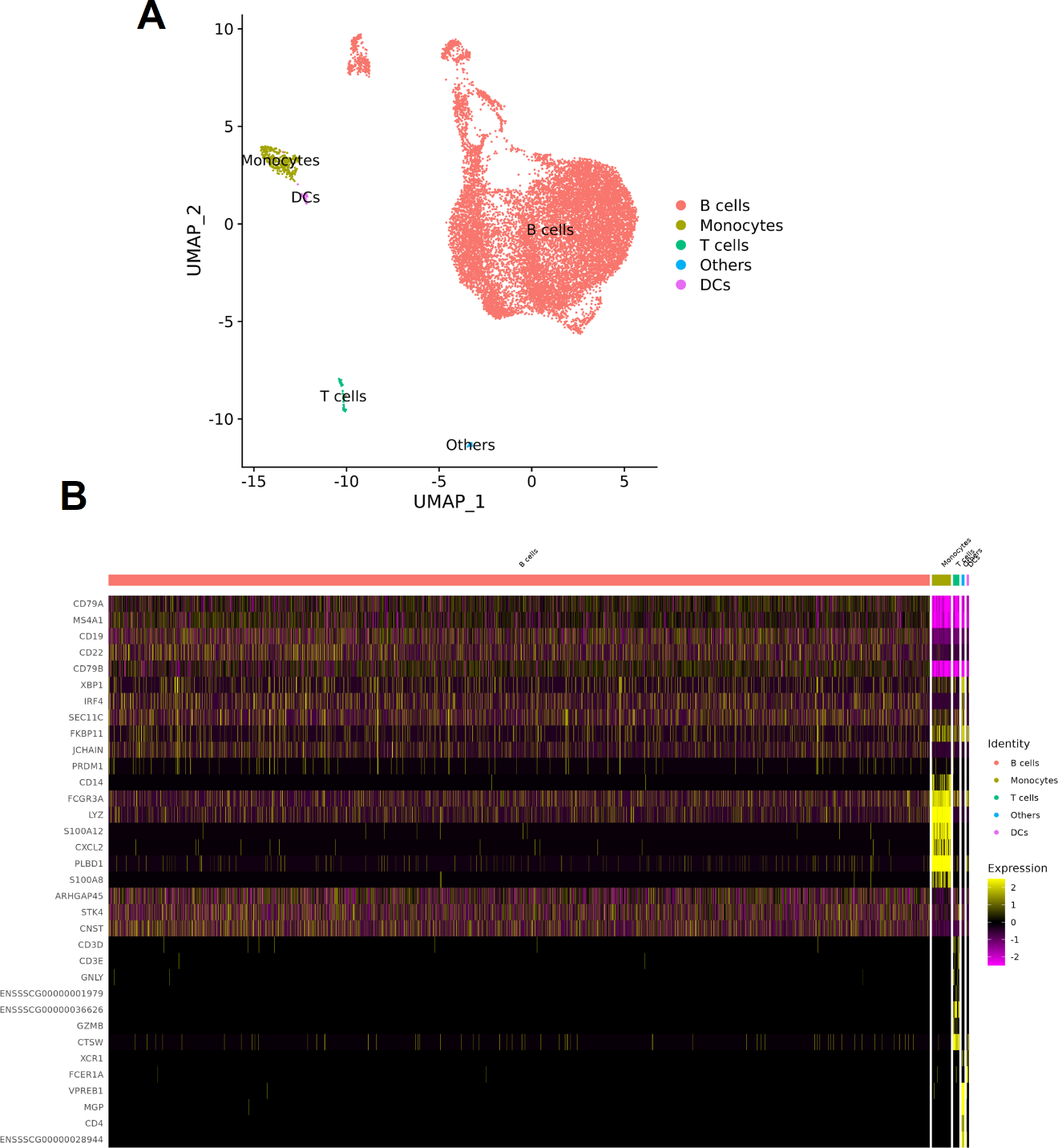


**Fig B. The phenotype and constitution of FMDV-binding cells revealed by scRNA-seq transcripts.** **(A)** UMAP plot of unsupervised clustering of FMDV-binding cells, comprising of majority B cells, as well as other minimal cell populations such as monocytes, T cell, dendritic cells (DCs) and unidentified cells. **(B)** Heatmap of differentially expressed genes in B cells, Monocytes, T cells, DCc, and others.


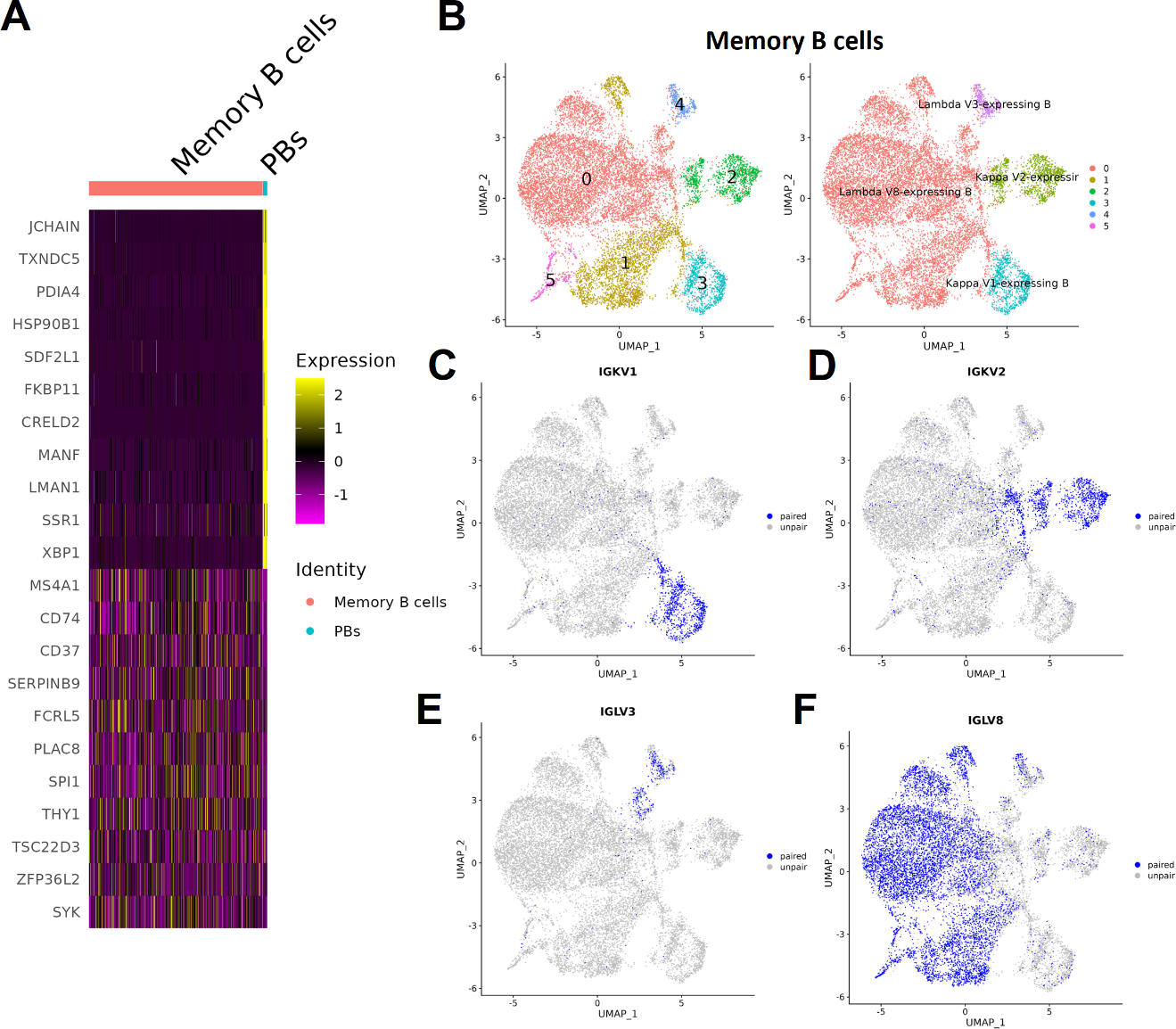


**Fig C. The heterogeneity of porcine memory B cells revealed by pairing analysis of BCR and transcripts.**  **(A)** Heatmap of differentially expressed genes between memory B cells and PBs. **(B-F)** UMAP plot of unsupervised clustering of porcine memory B cells, comprising of six clusters in **B**. According to the germline gene usages of VL, the memory B cells were separated into kappa V1-expressing B cells (pairing with cluster 3 in **C**), kappa V2-expressing B cells (pairing with cluster 2 in **D**), lambda V3-expressing B cells (pairing with cluster 4 in **E**) and lambda V8-expressing B cells (pairing with clusters 0, 1 and 5 in **F**).

**
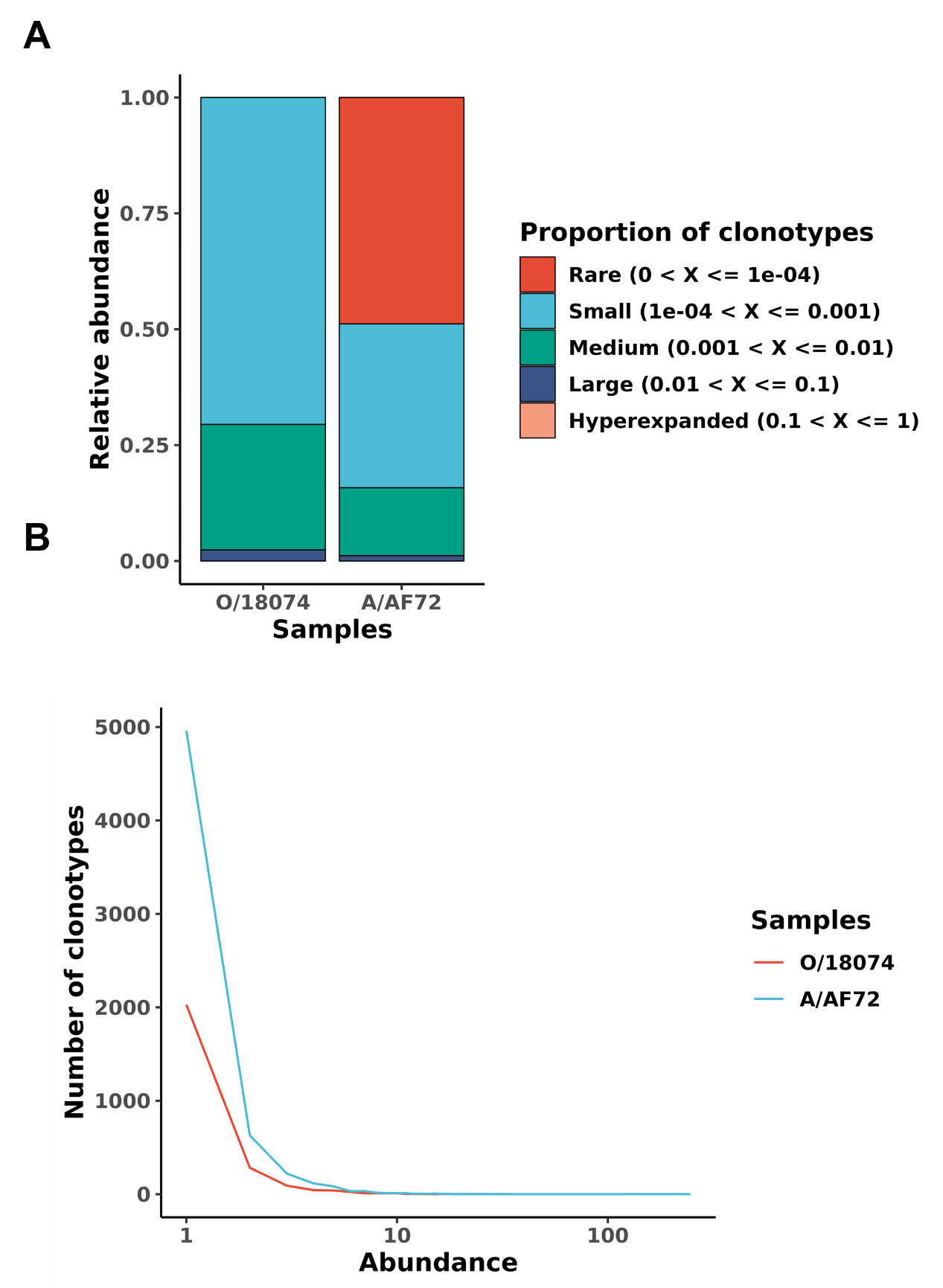
**

**Fig D. Analysis of the relative abundance and density of BCR clonotypes for serotype O and A specific BCR repertoires.** (A) The proportion of BCR clonotypes was shown in O/18074-specific and A/AF72-specific repertoires respectively. (B) The number of the clonotypes with different frequency indicated the difference in abundance between the two repertoires.

**
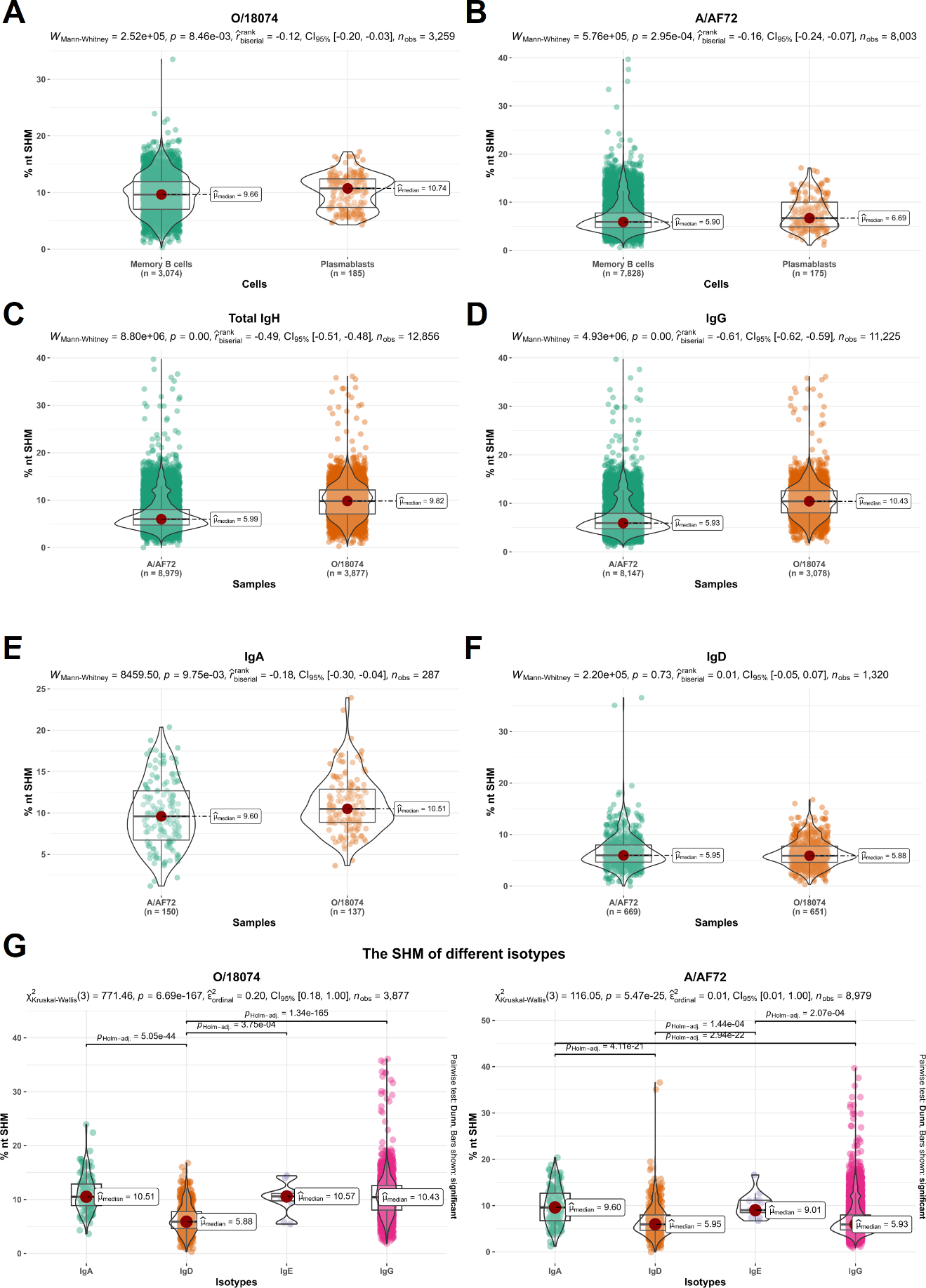
**

**Fig E. Analysis of the SHMs of porcine BCR repertoires.**

**(A)** The SHM difference of porcine VH between memory B cells and plasmablasts in FMDV serotype O-specific repertoire. **(B)** The SHM difference of porcine VH between memory B cells and plasmablasts in FMDV serotype A-specific repertoire. **(C-F)** The difference in SHM of each isotype antibodies between the serotype O and A specific BCR repertoires**.** The statistical analysis was performed using non-parametric Mann-Whitney test in R program and showed the medians in violin plot. **(G)** The difference in SHM among the isotypes (IgA, IgD, IgE and IgG) antibodies within each serotype O and A specific BCR repertoire. The statistical analysis was performed using Kruskal-Wallis chi-square test in R program and showed the medians in violin plot. P<0.05 indicates a significant difference between two samples. P<0.01 indicates a very significant difference between two samples. P<0.001 indicates an extremely significant difference between two samples. NS indicates no significant difference.


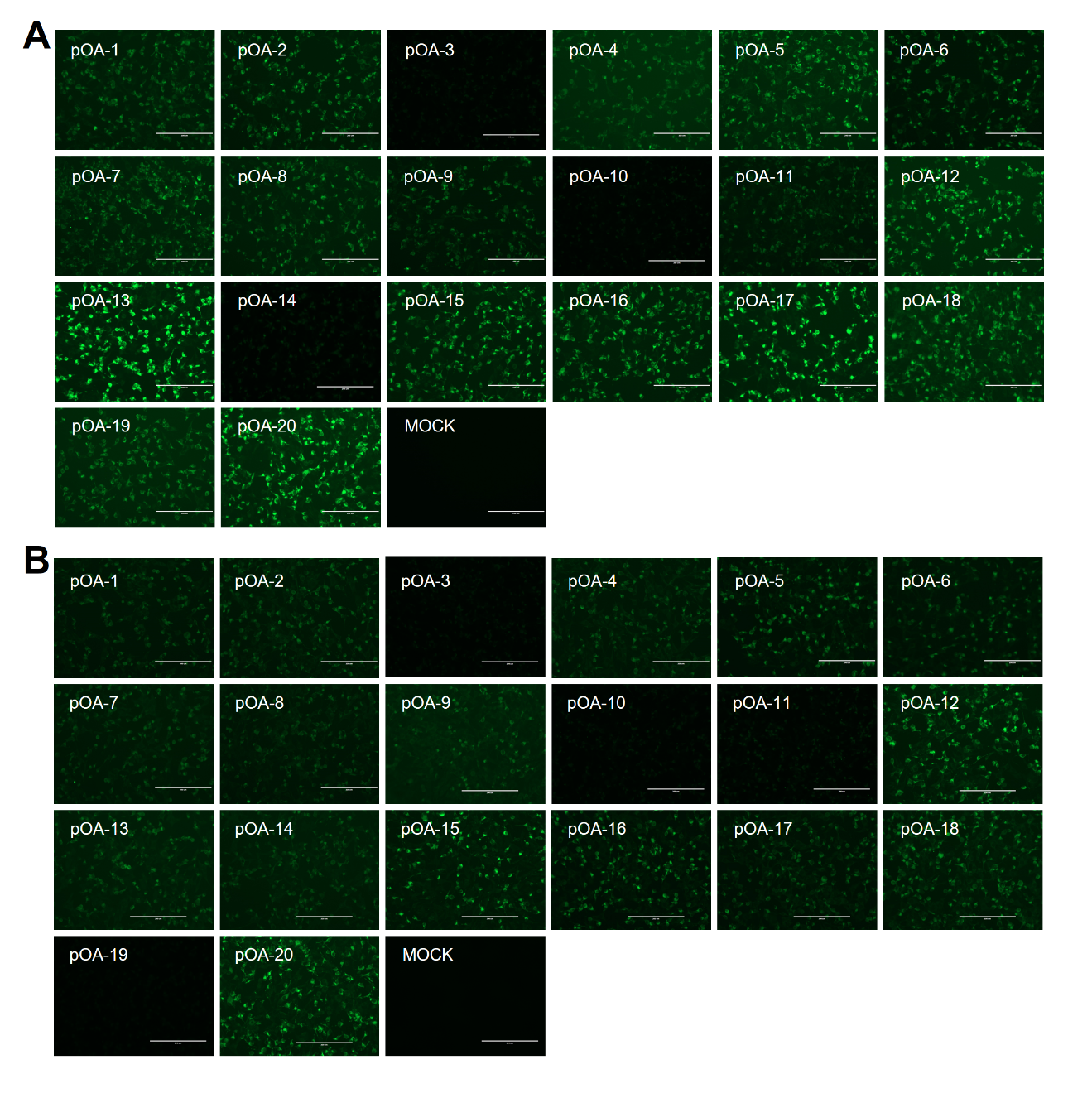


**Fig F. Identification of the reactivity of porcine mAbs with FMDV serotype O and A using indirect immunofluorescence assay (IFA). (A, B)** BHK-21 cells infected respectively with the O/18074 strain **(A)** or A/AF72 strain **(B)**, and the working concentration of the tested porcine mAbs was 5µg/ml, followed by incubation with rabbit anti-pig FITC (diluted 1:200 in PBS). The cells were observed under an EVOS® FL Imaging System (Life Technology, USA). The experiments were independently conducted in triplicate.


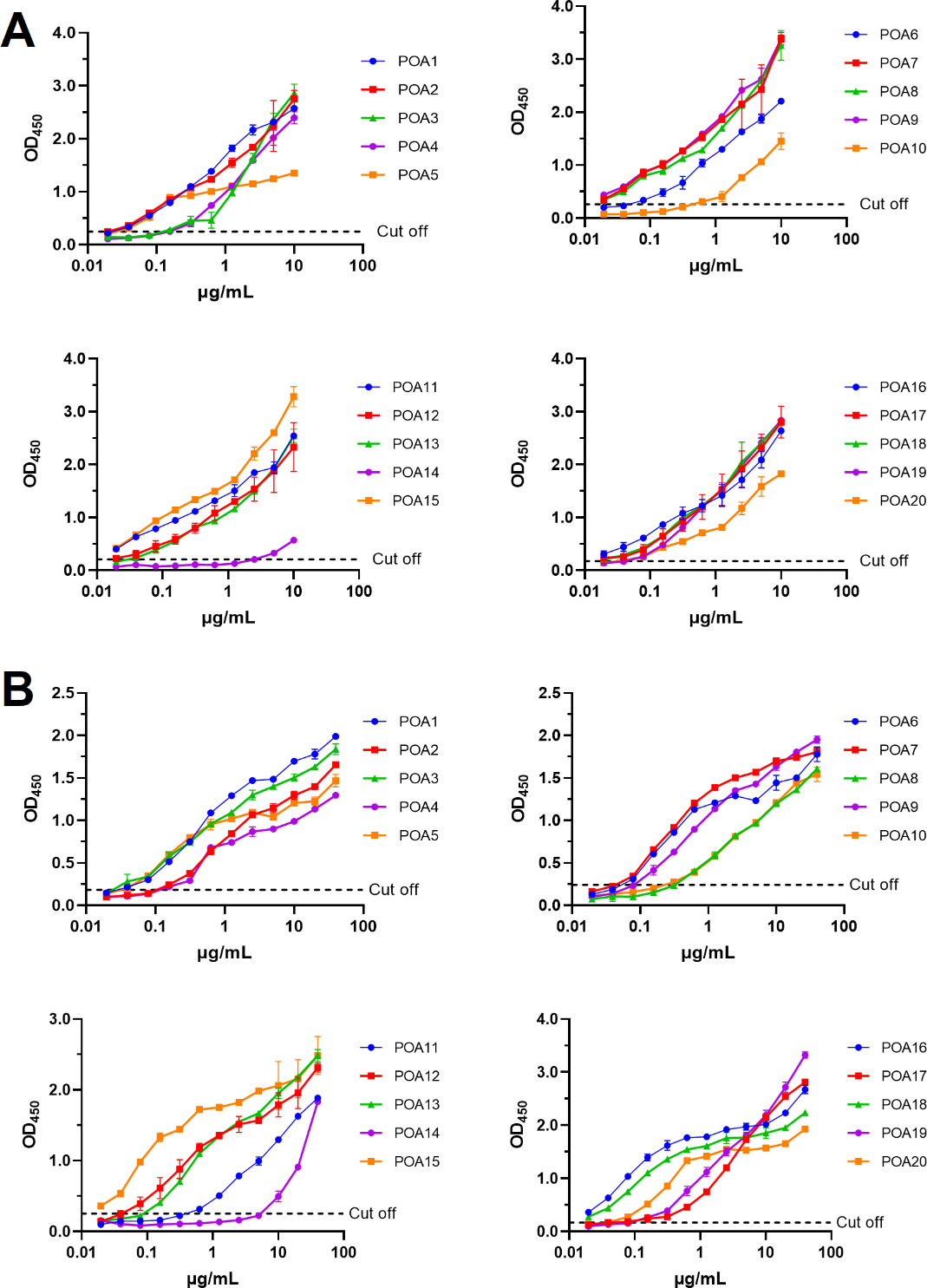


**Fig G. Identification of the reactivity of porcine mAbs with FMDV serotype O and A using enzyme-linked immunosorbent assay (ELISA). (A, B)** The ELISA plates were respectively coated with inactivated 146S antigen of O/Tibet/99 **(A)** or A/AF72 **(B)**, and then probed with different concentrations of 0-40µg/ml of the tested mAbs, followed by probing with HRP-conjugated goat anti-porcine IgG. Color was developed by adding 50 μl of TMB substrate (Pierce, Life Technology) for 10min at room temperature. The process was stopped by adding equal volumes of 1M H_2_SO_4_. Optical density at 405 nm (OD_450_) was measured on a microplate reader (Bio-Rad). The experiments were independently conducted in triplicate.


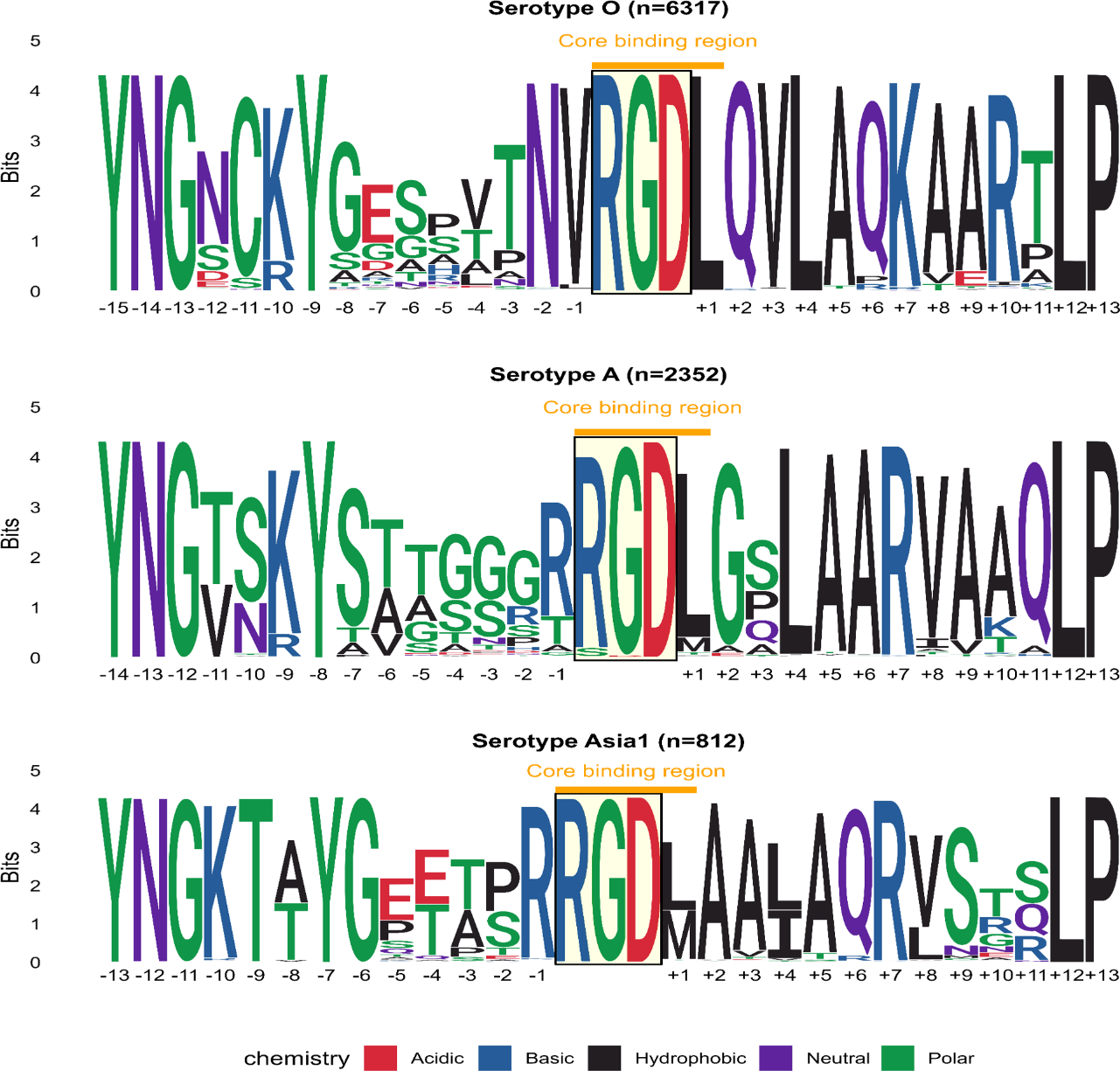


**Fig H. Analysis of sequence conservation of VP1 GH-loop of available FMDV strains in serotypes O, A and Asia1.** The full VP1 amino acids sequences of available FMDV serotype O, A and Asia1 were downloaded from national center for biotechnology information (NCBI) as of June 30, 2023. The sequence logo of VP1 GH-loop of FMDV serotype O (numbers of full VP1 sequences=6317), A (numbers of full VP1 sequences=2352) and Asia1 (numbers of full VP1 sequences=812). The key antigenic determinants on GH-loop that recognized by porcine bnAbs were marked with bold black line. The integrin receptor (αvβ6) recognition motif, “RGD”, was framed with rectangles and marked with yellow.


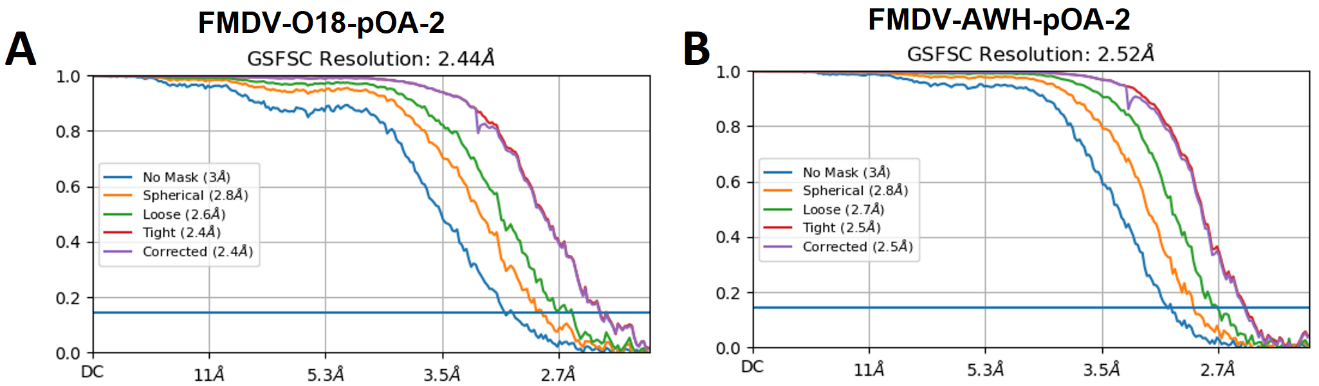


**Fig I. The resolution of the cryo-EM reconstruction complex.** (A, B) Fourier shell correlation (FSC) of FMDV-O18-POA2 complex (A) or FMDV-AWH-POA2 complex (B).

**
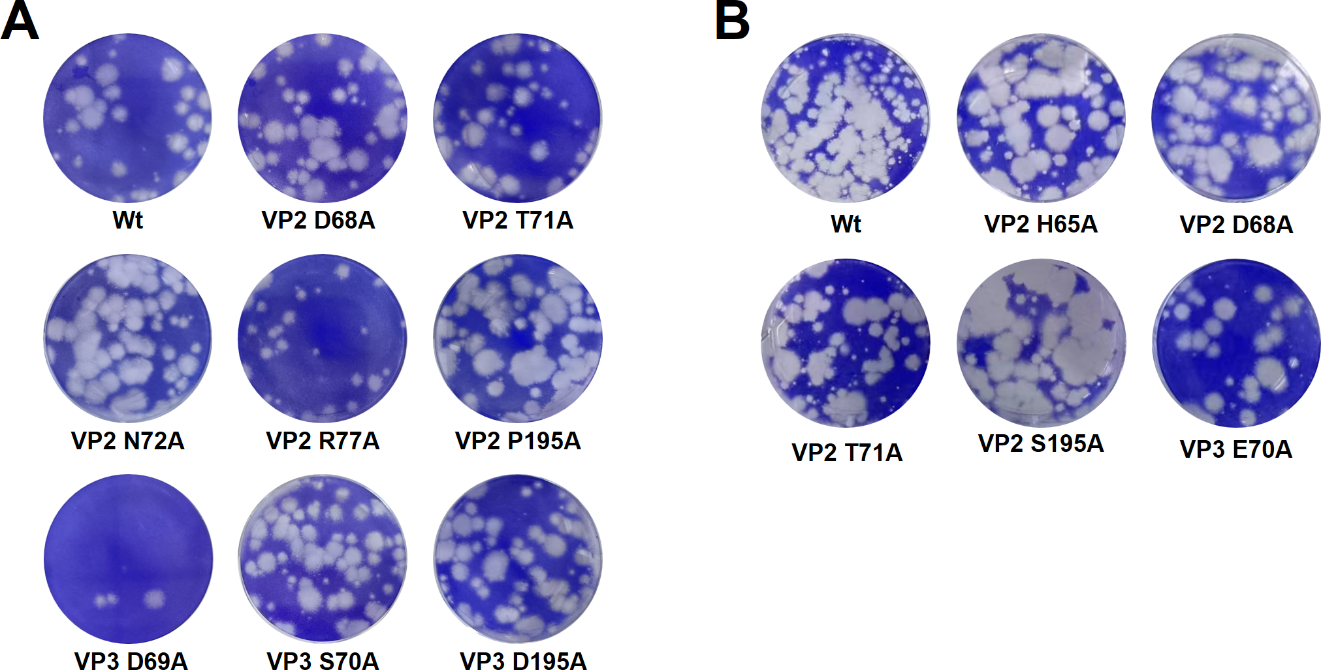
**

**Fig J. Identification of the rescued single-substitution mutants by plaque formation assay. (A)** The wild-type (O/18074) and rescued mutants (VP2 D68A, VP2 T71A, VP2 N72A, VP2 R77A, VP2 P195A, VP3 D69A, VP3 S70A and VP3 D195A) formed in BHK-21 cells, and the sizes were correlated to the CPE patterns. **(B)** The wild-type (A/WH/CHA/09 strain) and rescued mutants (VP2 H65A, VP2 D68A, VP2 T71A, VP2 P195A, VP3 S70A) formed in BHK-21 cells, and the sizes were correlated to the CPE patterns.


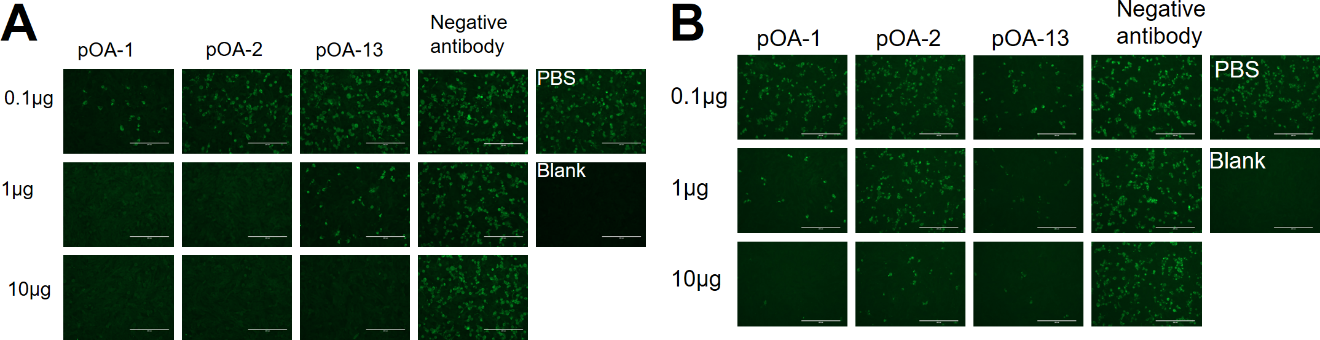


**Fig K.** **Neutralization mechanism of porcine bnAbs against FMDV serotype O and A.** The inhibition effect of porcine bnAbs on viral attachment to BHK-21 cells was determined by IFA. Different amounts of pOA-1, pOA-2 or pOA-13 were respectively mixed with FMDV serotype O (O/HN/CHA/93 strain) **(A)** or serotype A (A/WH/CHA/09 strain) **(B)** at 37°C for 1h, and the mixtures were loaded on BHK-21 cells at 4°C for 1h to allow virus attachment, then washed 3 times with cold PBS to remove unbound virus. The viruses were detected by IFA. The experiments were independently conducted in triplicate.


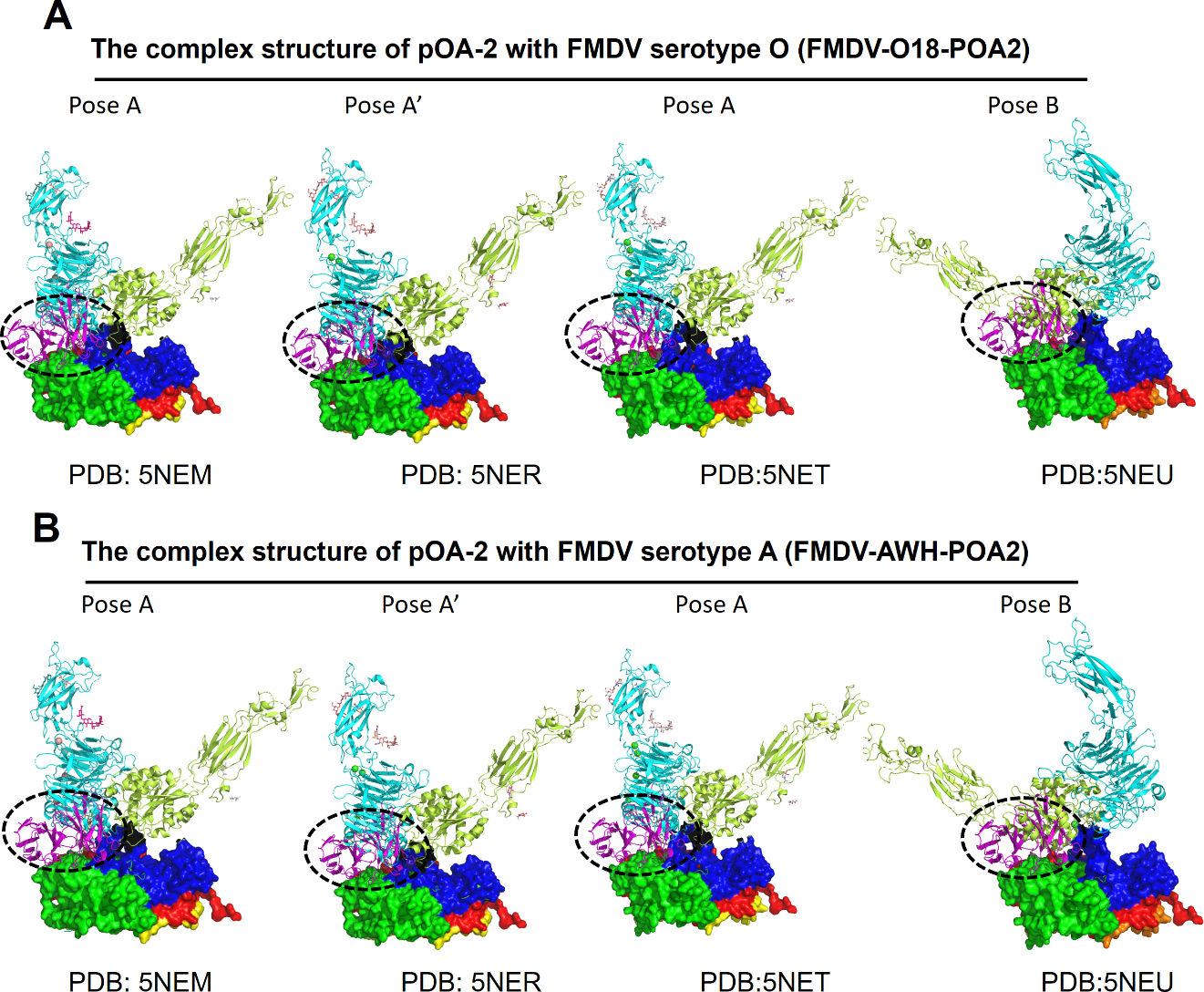


**Fig L. Binding modes of FMDV integrin receptor and antibody.** Binding modes of FMDV integrin receptor (avβ6) and bnAb pOA-2. (**A**) Superposition of FMDV- avβ6 with FMDV-O18-POA2 (**A**) and FMDV-AWH-POA2 (**B**). VP1, VP2, VP3 and VP4 of the protomer are shown in blue, green, red and yellow, respectively. The av and β6 chains of integrin (avβ6) and pOA-2 are drawn in cartoon representation and colored in cyan, limon and magenta, respectively. Black dashed circles show significant clashes between antibody (pOA-2) and integrin receptor.


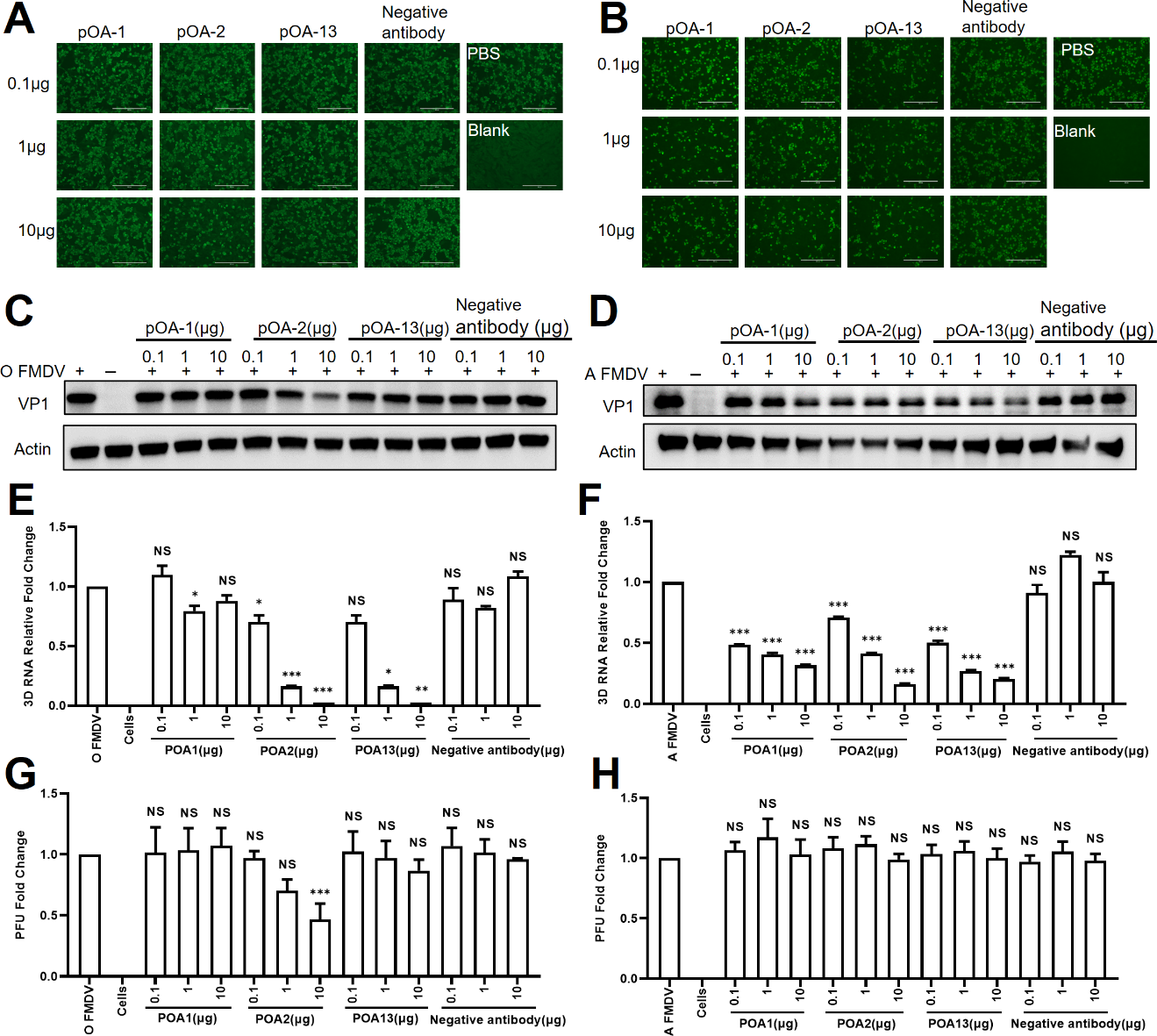


**Fig M. Effect of porcine bnAbs on virus at the post-attachment stage.** The BHK-21 cells were respectively incubated with FMDV serotype O (O/HN/CHA/93 strain) **(A, C, E, G)** or serotype A (A/WH/CHA/09 strain) **(B, D, F, H)** at 4°C for 1h. Subsequently, the cells were treated with different amounts of pOA-1, pOA-2 or pOA-13 at 37°C for 1h, the cells were washed with cold PBS to remove unbound virus, then the cells were further cultured for a duration of 4 hours. The viruses were quantified through detecting VP1 protein by IFA **(A, B)** and Western blotting **(C, D)**, 3D gene by qRT-PCR **(E, F)** and the numbers of PFUs by plaque phenotypes assay **(G, H)**. The experiments were independently conducted in triplicate. The data differences between conditions with virus only and different bnAb treatments were assessed using unpaired T-test (Holm-Sidak method, α = 0.05) in GraphPad Prism 7.2. *** Indicates an extremely significant difference at P<0.001. ** Indicates a very significant difference at P<0.01. * Indicates a significant difference at P<0.05. NS indicates no significant difference.


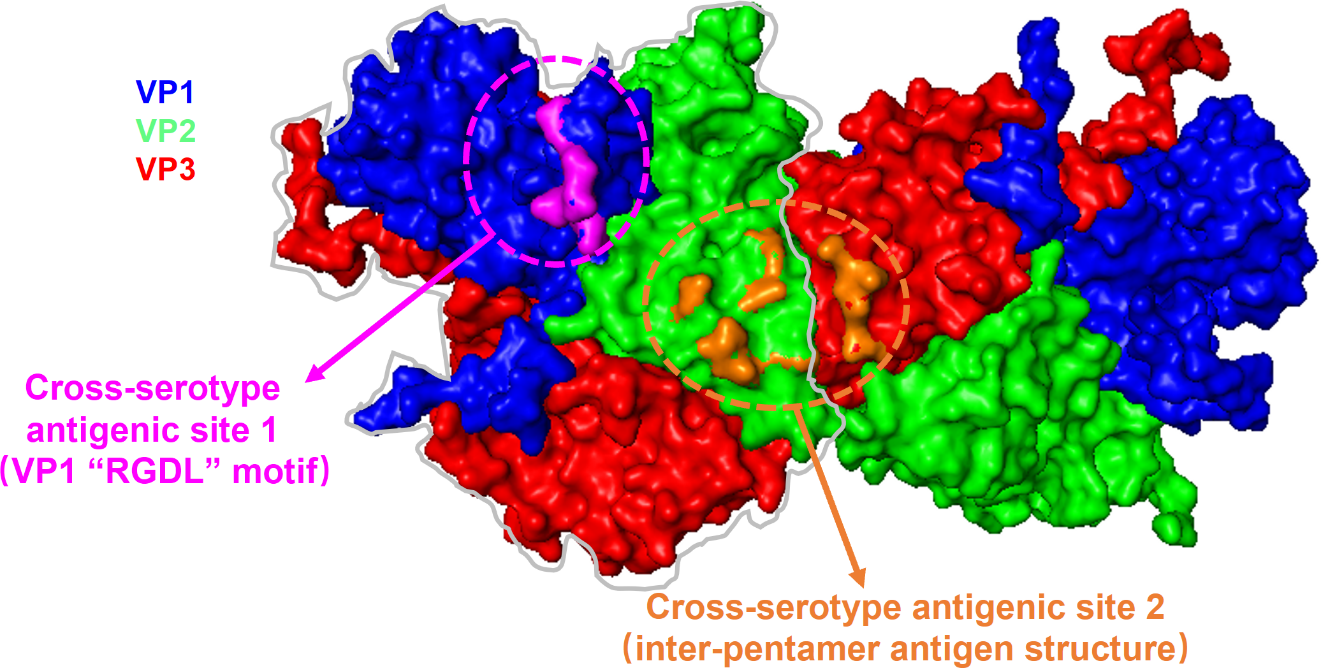


**Fig N. The recognized antigen structures of porcine cross-serotype bnAbs against FMDV.** Footprints of two distinct cross-serotype antigenic sites on surface of two protomers from two adjacent pentamers of FMDV. One protomer comprising of VP1, VP2, VP3 and VP4 was circled in grey line. Cross-serotype antigenic site 1 consisted of VP1 143, 145-148 and 151 position residues was marked in pink. Cross-serotype antigenic site 2 that consisted of VP2 65, 68, 71, 72, 77 and 195 position residues on one protomer and VP3 68, 69, 70 and 195 position residues on another protomer, was marked in orange.

**Table A to I**

**Table A. Porcine single B cell sequencing primers used for V(D)J amplifications.**

| V(D)J Amplification 1 | | |
| --- | --- | --- |
| Porcine B cell mix 1 | Forward primer | Reverse outer primers: |
|  | 5’-GATCTACACTCTTTCCCTACACGACGC-3’ | 5’-GGTCACTGRCTCGGGGAAGTAGC-3’  5’-CCTCCAGGTCACSGTCACG-3’  5’-TGTCCTTGCTGTCCTGCTCTG-3’  5’-TCCCGGACCAGGCAGGCG-3’  5’-CGGAGCTGGGCAGGAAGTC-3’  5’-GGTCACGGTCACTGGCTCTG-3’ |
| V(D)J Amplification 2 | | |
| Porcine B cell mix 2 | Forward primer | Reverse inner primers: |
|  | 5’-GATCTACACTCTTTCCCTACACGACGC-3’ | 5’-CAGGGGGCCAGAGGGTAGACC-3’  5’-GTCACTTATTAGACACACCAGGGTG-3’  5’-GATGAAGACGGATGGCTTGGC-3’  5’-CGCTGCTCCCCAGAGTCAGTG-3’  5’-GGGCCACCAGGCTCTCATCG-3’  5’-CTGACCAGGCAGCCCAGCG-3’ |

**Table B. The interactive residues between the integrin receptor (alpha V beta 6) with VP1 G-H loop on FMDV.**

| Receptor (alpha V beta 6) | FMDV VP1 | Distance | Interaction | Pose （A or A'） | PDB | Complex |
| --- | --- | --- | --- | --- | --- | --- |
| beta 6 K133 (NZ) | D46 (OD2) | 2.87 | H-bond | A | 5NEM | Panasia plus integrin Pose A |
| beta 6 K133 (OD2) | D46 (NZ) | 2.87 | salt bridge | A | 5NEM |  |
| alpha V D150 (OD1) | T142 (OG1) | 2.69 | H-bond | A | 5NEM |  |
| alpha V Y178 (OH) | N143 (O) | 2.74 | H-bond | A | 5NEM |  |
| alpha V Y178 (OH) | R145 (N) | 2.22 | H-bond | A | 5NEM |  |
| alpha V Y178 (OH) | R145 (O) | 2.68 | H-bond | A | 5NEM |  |
| alpha V F177 (O) | R145 (NH2) | 3.17 | H-bond | A | 5NEM |  |
| alpha V D218 (OD2) | R145 (NE) | 2.03 | salt bridge | A | 5NEM |  |
| beta 6 S122 (OG) | D147 (OD1) | 2.58 | H-bond | A | 5NEM |  |
| beta 6 S124 (OG) | D147 (OD1) | 3.17 | H-bond | A | 5NEM |  |
| beta 6 A123 (OG) | D147 (OD2) | 2.74 | H-bond | A | 5NEM |  |
| alpha V D150 (O) | T142 (OG1) | 2.9 | H-bond | A' | 5NER | Panasia plus integrin Pose A’ |
| alpha V D148 (OD2) | N143 (ND2) | 1.8 | H-bond | A' | 5NER |  |
| alpha V D218 (OD2) | R145 (NE) | 2 | salt bridge | A' | 5NER |  |
| beta 6 I216 (OG) | L148 (O) | 3.06 | H-bond | A' | 5NER |  |
| beta 6 K133 (NZ) | D46 (OD2) | 2.12 | H-bond | A | 5NET | O1M plus integrin Pose A |
| beta 6 K133 (OD2) | D46 (NZ) | 2.12 | salt bridge | A | 5NET |  |
| beta 6 S122 (OG) | D147 (OD1) | 2.42 | H-bond | A | 5NET |  |
| beta 6 A123 (OG) | D147 (OD2) | 3.31 | H-bond | A | 5NET |  |
| beta 6 A123 (O) | L151 (N) | 3.33 | H-bond | A | 5NET |  |
| alpha V Y178 (OH) | R145 (N) | 1.89 | H-bond | A | 5NET |  |
| alpha V Y178 (OH) | R145 (O) | 3.22 | H-bond | A | 5NET |  |
| alpha V D218 (OD2) | R145 (NE) | 2.22 | salt bridge | A | 5NET |  |
| beta 6 A105 (N) | D147 (OD2) | 2.74 | H-bond | B | 5NEU | O1M plus integrin Pose B |
| beta 6 N197 (N) | D147 (OD2) | 2.82 | H-bond | B | 5NEU |  |
| beta 6 A105 (O) | L151 (N) | 2.95 | H-bond | B | 5NEU |  |

The blue font refers to a hydrogen bond. The orange font refers to salt bridge.

**Table C. Porcine broad neutralizing mAb escape mutants.**

| MAb | Parent virus | Frequency  of mutants^$^ | Residue change | Neutralization  Concentration^#^(µg/ml) | Antigenic site |
| --- | --- | --- | --- | --- | --- |
| pOA-2 | O/HN/CHA/93 | 6/8 | VP2 D68N | 400 | Novel site |
|  |  | 1/8 | VP2 D68N; VP1 V15I | 400 |  |
|  |  | 1/8 | VP2 D68N; VP1 R133S; VP1 D138G | 400 |  |
| pOA-1 | O/HN/CHA/93 | 3/7 | VP1 Q149K | 400 | G-H loop |
|  |  | 1/7 | VP1 Q149R | 400 |  |
|  |  | 1/7 | VP1 R133S; VP1 D138A; VP1 Q149R | 400 |  |
|  |  | 1/7 | VP1 R133S; VP1 D138G; VP1 Q149R | 400 |  |
|  |  | 1/7 | VP1 K41R; VP1 T101A; VP1 Q149K | 400 |  |
| pOA-6 | O/HN/CHA/93 | 6/8 | VP1 L148R | 400 | G-H loop |
|  |  | 1/8 | VP1 H108R | 400 |  |
|  |  | 1/8 | VP1 R133S; VP1 S137D; VP1 D138A; VP1 L148R | 400 |  |
| pOA-7 | O/HN/CHA/93 | 4/6 | VP1 Q149R | 400 | G-H loop |
|  |  | 1/6 | VP1 Q149H | 400 |  |
|  |  | 1/6 | VP1 H108R | 400 |  |
| pOA-8 | O/HN/CHA/93 | 2/6 | VP1 Q149R | 400 | G-H loop |
|  |  | 1/6 | VP1 Q149K | 400 |  |
|  |  | 1/6 | VP1 S142N; VP1 Q149K | 400 |  |
|  |  | 1/6 | VP1 R133S; VP1 D138A | 400 |  |
|  |  | 1/6 | VP1 H108R | 400 |  |
| pOA-9 | O/HN/CHA/93 | 3/5 | VP1 Q149K | 400 | G-H loop |
|  |  | 1/5 | VP1 Q149L | 400 |  |
|  |  | 1/5 | VP1 H108R | 400 |  |
| pOA-13 | O/HN/CHA/93 | 6/8 | VP1 L148R | 400 | G-H loop |
|  |  | 1/8 | VP1 R133S; VP1 L148R | 400 |  |
|  |  | 1/8 | VP2 T64N; VP1 H108R; VP1 R133S; VP1 D138L; VP1 L148R | 400 |  |
| pOA-17 | O/HN/CHA/93 | 2/3 | VP1 L148R | 400 | G-H loop |
|  |  | 1/3 | VP1 K81R | 400 |  |
| pOA-19 | O/HN/CHA/93 | 4/8 | VP1 Q149R | 400 | G-H loop |
|  |  | 1/8 | VP1 Q149K | 400 |  |
|  |  | 1/8 | VP3 F209Y; VP1 Q149R | 400 |  |
|  |  | 1/8 | VP1 K81R; VP1 Q149R | 400 |  |
|  |  | 1/8 | VP1 V15I; VP1 Q149K | 400 |  |
| pOA-20 | O/HN/CHA/93 | 4/8 | VP1 L148R | 400 | G-H loop |
|  |  | 1/8 | VP2 T16I; VP2 A39T; VP1 L148R | 400 |  |
|  |  | 1/8 | VP1 A13T; VP1 L148R | 400 |  |
|  |  | 1/8 | VP1 A121I; VP1 L148R | 400 |  |
|  |  | 1/8 | VP1 A121S; VP1 L148R | 400 |  |

^#^Neutralization concentration was determined as the lowest antibody concentration that protected cells from CPE.

^$^Frequencies of the mutants are the number of mutants with the mutation at the indicated residue/total number of mutants obtained.

**Table D. Interface identification and interaction analysis of pOA-2 with FMDV O/18074 by PISA Program(5)**

| Interface | | | | | Interaction | | | | | | |
| --- | --- | --- | --- | --- | --- | --- | --- | --- | --- | --- | --- |
| O/18074 | **pOA-2** | | **BSA(Å^2^)^a^** | **Percentage^b^** | **Type^c^** | | **pOA-2^d^** | | **Dist.(Å)** | **O/18074^e^** | |
| 2: HIS65 | | H chain | 56.75 | \|\|\|\|\|\|\| |  | |  | |  |  | |
| 2: LEU66 | | H chain | 10.09 | \|\|\|\| |  | |  | |  |  | |
| 2: PHE67 | | H chain | 14.06 | \|\|\|\|\|\|\|\|\| |  | |  | |  |  | |
| 2:ASP68 | | H chain | 48.76 | \|\|\|\|\|\|\|\| | H  H | | H: LYS105[O]  H: LYS105[N] | | 3.74  3.40 | 2: ASP68[N]  2: ASP68[OD2] | |
| 2: GLY70 | | H chain | 4.18 | \|\|\| |  | |  | |  |  | |
| 2: ASN72 | | H chain | 67.21 | \|\|\|\|\|\| |  | |  | |  |  | |
| 2: SER74 | | H chain | 26.44 | \|\|\|\| |  | |  | |  |  | |
| 2: PHE75 | | H chain | 4.77 | \| |  | |  | |  |  | |
| 2: GLY76 | | H chain | 9.80 | \|\|\|\|\|\|\|\| |  | |  | |  |  | |
| 2: ARG77 | | H chain | 99.72 | \|\|\|\|\|\|\|\|\| | H  S  S | | H: GLN53[OE1]  H: GLU33[OE1]  H: GLU33[OE2] | | 2.77  3.71  3.70 | 2: ARG77[NH2]  2: ARG77[NH2]  2: ARG77[NH2] | |
| 2: CYS78 | | H chain | 5.32 | \|\| |  | |  | |  |  | |
| 2: HIS79 | | H chain | 17.88 | \|\|\|\| |  | |  | |  |  | |
| 2: GLU82 | | H chain | 0.74 | \| |  | |  | |  |  | |
| 2: CYS130 | | H chain | 11.80 | \|\| |  | |  | |  |  | |
| 2: SER131 | | H chain | 1.50 | \| |  | |  | |  |  | |
| 2: LYS134 | | H chain | 42.52 | \|\|\|\| |  | |  | |  |  | |
| 2: LYS198 | | H chain | 20.25 | \|\|\| |  | |  | |  |  | |
| 2: TYR200 | | H chain | 5.77 | \| |  | |  | |  |  | |
| 2:ASP68 | | L chain | 19.23 | \|\|\| | H | L: TYR51[OH] | | 2.78 | | 2: ASP68[OD2] |  |
| 2: GLY70 | | L chain | 6.02 | \|\|\|\|\| |  | |  | |  |  | |
| 2: THR71 | | L chain | 45.45 | \|\|\|\|\|\| | H  H | | L: TYR33[OH]  L: TYR33[OH] | | 3.07  3.33 | 2: THR71[N]  2: THR71[OG1] | |
| 2: ASN72 | | L chain | 68.21 | \|\|\|\|\| | H  H | | L: TYR92[OH]  L: ASN95[OD1] | | 3.27  2.29 | 2: ASN72[ND2]  2: ASN72[ND2] | |
| 2: SER74 | | L chain | 4.02 | \| |  | |  | |  |  | |
| 2: VAL189 | | L chain | 16.73 | \|\|\|\|\|\| |  | |  | |  |  | |
| 2: LYS190 | | L chain | 2.33 | \| |  | |  | |  |  | |
| 2:PRO195 | | L chain | 45.83 | \|\|\|\|\| | H | | L: TYR33[OH] | | 2.93 | 2: PRO195[O] | |
| 2: GLN196 | | L chain | 50.02 | \|\|\|\|\|\| |  | |  | |  |  | |
| 2: LYS198 | | L chain | 26.18 | \|\|\| |  | |  | |  |  | |
| C: SER70 | | H chain | 50.44 | \|\|\|\|\| | H | | H: THR102[O] | | 2.91 | C: SER70[OG] | |
| C: ARG72 | | H chain | 0.58 | \| |  | |  | |  |  | |
| C: THR135 | | H chain | 5.11 | \| |  | |  | |  |  | |
| C: THR68 | | L chain | 58.84 | \|\|\|\|\|\|\| | H  H | | L: ASN53[O]  L: SER54[OG] | | 2.93  2.43 | C: THR68[OG1]  C: THR68[O] | |
| C:ASP69 | | L chain | 65.88 | \|\|\|\|\|\|\| | S  S  S | | L: ARG55[NH1]  L: ARG55[NH2]  L: ARG55[NE] | | 3.85  3.16  2.89 | C: ASP69[OD2]  C: ASP69[OD2]  C: ASP69[OD2] | |
| C: SER70 | | L chain | 63.88 | \|\|\|\|\|\| | H  H  H | | L: ARG55[O]  L: TYR50[OH]  L: ARG55[O] | | 3.50  2.65  3.67 | C: SER70[N]  C: SER70[OG]  C: SER70[OG] | |
| C:ASP71 | | L chain | 15.71 | \|\| |  | |  | |  |  | |
| C:ASP195 | | L chain | 16.05 | \|\| | H | | L: ASN53[ND2] | | 3.42 | C: ASP195[OD2] | |

a BSA: Buried Surface Area; b ||||: Buried area percentage, one bar per 10%. c H, Hydrogen bond; S, Salt bridge. d: Interactive atoms of amino acid side chain of pOA-2. e: Interactive atoms of amino acid side chain of O/18074.

**Table E. Interface identification and interaction analysis of pOA-2 with FMDV A/WH/CHA/09 by PISA Program(5)**

| Interface | | | | | Interaction | | | |
| --- | --- | --- | --- | --- | --- | --- | --- | --- |
| A/WH/CHA/09 | **pOA-2** | | **BSA(Å^2^)^a^** | **Percentage^b^** | **Type^c^** | **pOA-2^d^** | **Dist.(Å)** | **A/WH/CHA/09^e^** |
| 2: LYS64 | | H chain | 3.99 | \| |  |  |  |  |
| 2: HIS65 | | H chain | 52.34 | \|\|\|\|\|\|\| | H | H: LYS105[NZ] | 3.11 | 2: HIS65[O] |
| 2: LEU66 | | H chain | 17.85 | \|\|\|\|\|\|\|\| |  |  |  |  |
| 2: PHE67 | | H chain | 12.72 | \|\|\|\|\|\|\|\|\|\| |  |  |  |  |
| 2:ASP68 | | H chain | 34.19 | \|\|\|\|\|\|\|\| | H  H | H: LYS105[O]  H: LYS105[N] | 3.15  2.99 | 2: ASP68[N]  2: ASP68[OD2] |
| 2: THR70 | | H chain | 15.60 | \|\|\|\|\| |  |  |  |  |
| 2: ASP72 | | H chain | 51.57 | \|\|\|\|\| | H  S  S | H: HIS108[NE2]  H: HIS108[NE2]  H: HIS108[NE2] | 2.65  2.65  3.86 | 2: ASP72[OD1]  2: ASP72[OD1]  2: ASP72[OD2] |
| 2: LYS73 | | H chain | 26.05 | \|\|\|\|\|\|\|\| |  |  |  |  |
| 2: PRO74 | | H chain | 39.98 | \|\|\|\|\| |  |  |  |  |
| 2: PHE75 | | H chain | 7.97 | \|\| |  |  |  |  |
| 2: GLY76 | | H chain | 5.48 | \|\|\|\|\|\|\| |  |  |  |  |
| 2: HIS77 | | H chain | 48.36 | \|\|\|\|\|\|\|\|\| |  |  |  |  |
| 2: ILE78 | | H chain | 4.88 | \|\| |  |  |  |  |
| 2: GLU79 | | H chain | 26.40 | \|\|\|\|\|\| |  |  |  |  |
| 2: LYS80 | | H chain | 8.50 | \| |  |  |  |  |
| 2: GLU131 | | H chain | 33.85 | \|\|\| |  |  |  |  |
| 2: PHE132 | | H chain | 5.28 | \|\| |  |  |  |  |
| 2: LYS137 | | H chain | 28.05 | \|\|\|\|\|\|\|\| | H  S | H: ASP56[OD2]  H: ASP56[OD2] | 2.86  2.86 | 2: LYS137[NZ]  2: LYS137[NZ] |
| 2: GLN196 | | H chain | 0.29 | \| |  |  |  |  |
| 2: LYS198 | | H chain | 16.05 | \|\| | H | H: GLY103[O] | 3.67 | 2: LYS198[NZ] |
| 2:ASP68 | | L chain | 13.78 | \|\|\| | H | L: TYR51[OH] | 2.95 | 2:ASP68[OD2] |
| 2: THR70 | | L chain | 16.13 | \|\|\|\|\|\| |  |  |  |  |
| 2: THR71 | | L chain | 42.76 | \|\|\|\|\|\| | H  H | L: TYR33[OH]  L: TYR33[OH] | 3.09  3.36 | 2: THR71[N]  2: THR71[OG1] |
| 2: ASP72 | | L chain | 58.43 | \|\|\|\|\|\| | H | L: ASN95[ND2] | 2.93 | 2: ASP72[O] |
| 2:PRO74 | | L chain | 9.87 | \|\| |  |  |  |  |
| 2: THR189 | | L chain | 17.55 | \|\|\|\| |  |  |  |  |
| 2: SER195 | | L chain | 50.28 | \|\|\|\|\|\| | H  H | L: THR31[N]  L: TYR33[OH] | 3.58  3.29 | 2: SER195[OG]  2: SER195[O] |
| 2: GLN196 | | L chain | 42.38 | \|\|\|\|\|\| |  |  |  |  |
| 2: LYS198 | | L chain | 13.67 | \|\| |  |  |  |  |
| C: GLU70 | | H chain | 32.98 | \|\|\| |  |  |  |  |
| C: ALA68 | | L chain | 37.21 | \|\|\|\| |  |  |  |  |
| C: ASP69 | | L chain | 40.90 | \|\|\|\|\| | H  S | L: ARG55[NH1]  L: ARG55[NH1] | 3.54  3.54 | C: ASP69[OD1]  C: ASP69[OD1] |
| C: GLU70 | | L chain | 80.81 | \|\|\|\|\|\| |  |  |  |  |

a BSA: Buried Surface Area; b ||||: Buried area percentage, one bar per 10%. c H, Hydrogen bond; S, Salt bridge. d: Interactive atoms of amino acid side chain of pOA-2. e: Interactive atoms of amino acid side chain of A/WH/CHA/09.

**Table F. FMDV O/18074 with pOA-2 interaction residues.**

| Domain | Residue | Distance(Å) | pOA-2 | CDR |
| --- | --- | --- | --- | --- |
| **Protomer1**  **VP2 βB** | H65(O) | 3.5 | K105(NZ) | HCDR3 |
|  | L66(O) | 3.6 | K105(CD) | HCDR3 |
|  | F67(CA) | 3.8 | K105(CB) | HCDR3 |
|  | D68(N) | 3.7 | K105(O) | HCDR3 |
|  | D68(OD2) | 3.3 | K105(N) | HCDR3 |
|  | D68(OD2) | 2.9 | Y51(OH) | LCDR2 |
|  | G70(CA) | 3.6 | Y33(OH) | LCDR1 |
| **Protomer1**  **VP2 BC-loop** | T71(N) | 3.1 | Y33(OH) | LCDR1 |
|  | T71(OG1) | 3.3 | Y33(OH) | LCDR1 |
|  | N72(ND2) | 2.9 | R107(NH1) | HCDR3 |
|  | N72(ND2) | 3.5 | Y92(OH) | LCDR3 |
|  | N72(ND2) | 2.3 | N95(OD1) | LCDR3 |
|  | F75(O) | 3.5 | F57(CZ) | HCDR2 |
| **Protomer1**  **VP2 βC** | R77(NH2) | 3.7 | E33(OE1) | HCDR1 |
|  | R77(NH2) | 3.7 | E33(OE2) | HCDR1 |
|  | R77(NH1) | 3.4 | Q53(OE1) | HCDR2 |
|  | R77(NH2) | 2.8 | Q53(OE1) | HCDR2 |
| **Protomer1**  **VP2 HI-loop** | P195(O) | 2.9 | Y33(OH) | LCDR1 |
|  | Q196(OE1) | 3.5 | Y33(CE1) | LCDR1 |
|  | Q196(OE1) | 3.6 | Y51(CD1) | LCDR2 |
| **Protomer1**  **VP2 βI** | K198(CE) | 3.2 | G103(O) | HCDR3 |
| **Protomer2**  **VP3 BC-loop** | T68(OG1) | 3.0 | N53(O) | LCDR2 |
|  | T68(O) | 2.5 | S54(OG) | L-FR3 |
|  | D69(OD2) | 3.0 | R55(NE) | L-FR3 |
|  | D69(OD2) | 3.9 | R55(NH1) | L-FR3 |
|  | D69(OD2) | 3.2 | R55(NH2) | L-FR3 |
|  | S70(OG) | 2.6 | Y50(OH) | L-FR2 |
|  | S70(OG) | 2.9 | T102(O) | HCDR3 |
| **Protomer2**  **VP3 HI-loop** | D195(OD2) | 3.4 | N53(ND2) | LCDR2 |

The interaction residues were computed using the CCP4(6) hydrogen bond distance cutoff of 4.0 Å and the salt-bridge distance cutoff of 4.0 Å. The blue font refers to a hydrogen bond. The orange font refers to salt bridge.

**Table G. FMDV A/WH/CHA/09 with pOA-2 interaction residues.**

| **Domain** | **Residue** | **Distance(Å)** | **pOA-2** | **CDR** |
| --- | --- | --- | --- | --- |
| **Protomer1**  **VP2 βB** | H65(O) | 3.1 | K105(NZ) | HCDR3 |
|  | L66(O) | 3.1 | K105(CE) | HCDR3 |
|  | D68(N) | 3.1 | K105(O) | HCDR3 |
|  | D68(OD2) | 3.0 | K105(N) | HCDR3 |
|  | D68(OD2) | 2.9 | Y51(OH) | LCDR2 |
|  | T70(CB) | 3.3 | Y33(OH) | LDCR1 |
| **Protomer1**  **VP2 BC-loop** | T71(N) | 3.1 | Y33(OH) | LCDR1 |
|  | T71(OG1) | 3.4 | Y33(OH) | LCDR1 |
|  | D72(O) | 2.9 | N95(ND2) | LCDR3 |
|  | D72(OD1) | 2.7 | H108(NE2) | HCDR3 |
|  | D72(OD2) | 3.9 | H108(NE2) | HCDR3 |
|  | G76(C) | 3.7 | F57(CZ) | HCDR2 |
| **Protomer1**  **VP2 EF-loop** | E131(OE2) | 3.6 | T54(CG2) | HCDR2 |
| **Protomer1**  **VP2 αB** | K137(NZ) | 2.9 | D56(OD2) | HCDR2 |
| **Protomer1**  **VP2 HI-loop** | S195(OG) | 3.6 | T31(N) | LCDR1 |
|  | S195(O) | 3.3 | Y33(OH) | LCDR1 |
|  | Q196(NE2) | 3.0 | Y51(CE2) | LCDR2 |
| **Protomer1**  **VP3 BC-loop** | D69(OD1) | 3.5 | R55(NH1) | L-FR3 |
|  | E70(CG) | 3.0 | Y50(OH) | L-FR2 |
|  | E70(OE2) | 2.0 | S54(OG) | L-FR3 |

The interaction residues were computed using the CCP4(6) hydrogen bond distance cutoff of 4.0 Å and the salt-bridge distance cutoff of 4.0 Å. The blue font refers to a hydrogen bond. The orange font refers to salt bridge.

**Table H. Sequence identity of pOA-2 epitopes in FMDV serotype O and A**

|  |  |  |  | Percent identity by serotype (%) |  |
| --- | --- | --- | --- | --- | --- |
| Position | **Consensus^a^** | **O/18074**  **sequence** | **A/CHA/WH/09**  **sequence** | **Serotype O**  **(VP2-1581/VP3-1496)^b^** | **Serotype A**  **(VP2-852/VP3-777) ^b^** |
| VP2 65 | H (87.3%) | H | H | H (99.8) | H (64.1), Y (21.7), F (13.8) |
| VP2 66 | L (99.5%) | L | L | L (99.9) | L (98.7) |
| VP2 67 | F (99.9%) | F | F | F (99.9) | F (99.9) |
| VP2 68 | D (99.3%) | D | D | D (99.9) | D (98.1) |
| VP2 70 | V (63.3%) | G | T | V (97.3) | T (100.0) |
| VP2 71 | T (93.1%) | T | T | T (99.9) | T (80.6) |
| VP2 72 | S (61.2%) | N | D | S (94.2) | D (95.8) |
| VP2 75 | F (100.0%) | F | F | F (100.0) | F (100.0) |
| VP2 77 | R/H (97.5%) | R | H | R (98.9) | H (95.1) |
| VP2 131 | S (64.4%) | S | E | S (99.1) | E (74.8), D (11.7), K (9.5) |
| VP2 137 | L (62.2%) | L | K | L (95.8) | K (99.5) |
| VP2 195 | P (64.7%) | P | S | P (99.6) | T (46.4), A (28.4), S (13.6) |
| VP2 196 | Q (99.0%) | Q | Q | Q (99.6) | Q (97.4) |
| VP2 198 | K(100%) | K | K | K(100%) | K(100%) |
| VP3 68 | T (97.6%) | T | A | T (98.9) | T (95.0) |
| VP3 69 | D (95.9%) | D | D | D (98.6) | D (90.7) |
| VP3 70 | S (65.0%) | S | E | S (98.7) | E (60.1), D (34.1) |
| VP3 195 | D (52.2%) | D | A | D (79.3), E (17.2) | D (99.4) |
| Avg^c^ | 83.5 |  |  | 92.2 | 88.6 |

isolates

^a^ Most common residue at position across all serotype O and A sequences.

^b^ Number of sequences available for FMDV in the NCBI at the time of download on June 30, 2023.

^c^ Average conservation of most common residue both serotype O and A.

**Table I. Cryo-EM data collection and refinement statistics**

|  | FMDV-O18-POA2 | FMDV-AWH-POA2 |
| --- | --- | --- |
| Data collection and processing |  |  |
| Magnification | 105,000 | 105,000 |
| Voltage (kV) | 300 | 300 |
| Electron exposure (e^–^/Å^2^) | 60 | 60 |
| Defocus range (μm) | -2.0 to -0.8 | -2.0 to -0.8 |
| Pixel size (Å) | 0.83 | 0.83 |
| Symmetry imposed | I1 | I1 |
| Final particle images (no.) | 3202 | 7341 |
| Map resolution (Å) | 2.44 | 2.52 |
| Map sharpening B factor (Å^2^) | -50.7 | -80.2 |
| Model Building and validation |  |  |
| Initial model used (PDB code) | 7D3K | 7D3R |
| MolProbity score | 1.55 | 1.34 |
| Clash score | 5.24 | 3.33 |
| Rotamer outliers (%) | 0.00 | 0.00 |
| R.M.S.D. of bond lengths (Å) | 0.004 | 0.005 |
| R.M.S.D. of angles (∘) | 0.581 | 0.561 |
| Favored (%) | 96.09 | 96.61 |
| Allowed (%) | 3.68 | 3.28 |
| Outliers (%) | 0.23 | 0.11 |

**SI References**

1. Reimold AM, Iwakoshi NN, Manis J, Vallabhajosyula P, Szomolanyi-Tsuda E, Gravallese EM, et al. Plasma cell differentiation requires the transcription factor XBP-1. Nature. 2001;412(6844):300-7.

2. Chong AS, Ansari MJ. Heterogeneity of memory B cells. American journal of transplantation : official journal of the American Society of Transplantation and the American Society of Transplant Surgeons. 2018;18(4):779-84.

3. Weisel NM, Joachim SM, Smita S, Callahan D, Elsner RA, Conter LJ, et al.Surface phenotypes of naive and memory B cells in mouse and human tissues. Nature immunology. 2022;23(1):135-45.

4. Li K, He Y, Wang L, Li P, Wang S, Sun P, et al. Two Cross-Protective Antigen Sites on Foot-and-Mouth Disease Virus Serotype O Structurally Revealed by Broadly Neutralizing Antibodies from Cattle. Journal of virology. 2021;95(21):e0088121.

5. Krissinel E, Henrick K. Inference of macromolecular assemblies from crystalline state. J Mol Biol. 2007;372(3):774-97.

6. Winn MD, Ballard CC, Cowtan KD, Dodson EJ, Emsley P, Evans PR, et al. Overview of the CCP4 suite and current developments. Acta crystallographica Section D, Biological crystallography. 2011;67(Pt 4):235-42.
