# Supplementary material for: Discovery, recognized antigenic structures, and evolution of cross-serotype broadly neutralizing antibodies from porcine B-cell repertoires against foot-and-mouth disease virus
Source: PLoS Pathog. 2024 Oct 15;20(10):e1012623. doi: 10.1371/journal.ppat.1012623 (PMC11508087; doi:10.1371/journal.ppat.1012623)
Supplement: Attachment — Submitted filename: Response to comments.docx [file ppat.1012623.s004.docx]

Dear editors and reviewers:

Thank you for your letter and the reviewers' comments on our manuscript titled "Discovery, evolution, and recognized antigenic structures of cross-serotype broadly neutralizing antibodies from porcine B-cell repertoires against foot-and-mouth disease virus". These comments are highly valuable and have greatly contributed to the revision and improvement of our paper. They also provide important guidance for our research. We have thoroughly reviewed the comments and made necessary corrections to address them appropriately. All figures have been carefully examined and adjusted using PACE digital diagnostic tool. Revised supplementary data has been highlighted in yellow within the supporting information document. The main corrections in the paper along with our responses to editors' and reviewers' comments are outlined below.

Best wishes

Kun Li

Reviewer's Responses to Questions

Part I - Summary
Please use this section to discuss strengths/weaknesses of study, novelty/significance, general execution and scholarship.

Reviewer #1: A single mini pig was immunised with a prime and boost of a single O serotype vaccine antigen. Approximately 4 months later, the pig was immunised with two different O serotype antigens and two serotype A antigens. Neutralising antibody titres generally developed at the expected times post immunisation (O-specific responses developed after immunisation with O antigens and A-specific responses only developed after the later immunisation with A antigen). Interestingly, after the immunisation with A antigens, a neutralising response also developed against a third serotype, Asia1.

B cells from PBMCs harvested after the final immunisation were enriched, separated by specificity to O or A serotype and B cell receptor repertoires characterised. Clonotypes shared between the O- and A-specific repertoires were identified, expressed as recombinant mabs and found to be reactive with O and A and also with Asia1.

Somatic hypermutation (SHM) and clonal evolution was investigated for several antibody lineages containing broadly neutralising antibody clonotypes. From one lineage containing broadly neutralising antibody pOA-2, the authors propose a model where the most broadly neutralising antibodies may be lost due to further SHM which selects for increasing potency of neutralisation at the expense of breadth.

The structures of mab pOA-2 interacting with FMDV serotypes O and A are solved showing the interaction is across two pentamers. The antibody interaction is slightly different between O and A serotypes but with some conserved features, especially the involvement of VP2 D68 which was found to be critically required for ab binding and which is very highly conserved across O, A , Asia1 serotypes.

The other nine broadly neutralising antibodies characterised were all found to react with a linear epitope which mapped to the G-H loop, with the RGDL integrin-binding motif forming the core of the epitope. The G-H loop residues involved in interactions with integrin are proposed to be the same as those targeted by the broadly neutralising mabs.

Generation of escape mutants against the antibodies and single site viral reverse genetics showed that one or both of RGD +1 and RGD+2 appeared to be the most important residues required for antibody neutralisation of both serotypes. For three mabs, the kinetics of binding to virus was determined and the ability of mabs to prevent attachment to cells or entry into cells was investigated (some of this questionable). Mab pOA-2 appears to destabilise the virus.

This is a lot of work with some interesting findings and should certainly be published.

In my opinion this paper includes too much data and could have been broken up into at least two separate papers! Supplementary material should supplement the main results (e.g. provide additional detail) but should not be required to understand the main story. In this paper, the reader is constantly referred to the supplementary material and it is often not clear whether this is part of the main story or a less important ‘supplementary’ part of the story. I may not have reviewed in detail all of the supplementary material.

I found some parts of the text in the results section associated with figures 1 and 2 were confusing and could perhaps be written more clearly. I have given some examples and suggestions for extra clarity throughout the manuscript in the minor issues section.

For some of the work investigating frequency of SHM I felt the findings and their significance could be better explained. However, the results section associated with fig 3 seemed clearly explained and was a strong point.

My biggest recommendation for this paper is to explain the simple/conventional parts of the study first (i.e. immunisation schedule currently Fig 2D and the time course of serotype specific polyclonal response currently Fig 2E). This would make everything else much easier to understand.

The structural biology seemed convincing to me and the discovery of a bnAb able to bind across pentamers of multiple serotypes with a destabilising mechanism is interesting and a strong point of the study.

The GH loop is immunodominant and presumably it was therefore expected to identify many GH reactive antibodies? However I was surprised that so many RGD binding antibodies could be cross reactive between serotypes. Was this expected? How do these findings sit with existing knowledge of GH loop reactive monoclonals? Is this a special feature of broadly neutralising mabs?

The binding studies and IF are weak. There is so much data in this paper, is this even required?

Response:

Thanks very much for the advice. These comments are valuable and contributed great for improvement of the manuscript. The immunization schedule and serotype specific polyclonal response in Figure 2D-E were moved to Figure 1. Given to that the levels of SHMs were affected by many factors (Such as allelic variation in Ig genes between individual pigs, the under-sampling of germline Ig gene segments in pig, and the immunization times with serotype O and A), parts of data on SHMs in Figure 1 were moved to supplemental files. We reorganized the results sections following the orders of discovery, recognized antigen structure, antibody evolution, and the neuralization mechanism.

Yes, the G-H loop is immunodominant. In previous reports, lots of neutralization antibodies binding G-H loop are serotype specific. Obtainment of many cross-reactive antibodies targeting the G-H loop in this study also surprised us. Additionally, we have also identified that the O-specific neutralizing monoclonal antibodies (mAbs) recognizing linear epitopes exhibit significant overlap with those bound by the cross-serotype bnAbs. We therefore speculated that induction of cross-serotype neutralization antibodies was determined by at least three factors: the conformational flexibility of G-H loop on viral particle, porcine breed, and the sequential immunization schedule. The dynamic of cross-serotype neutralization production will be further surveyed in future study.

The binding ability is weak, as observed in IF for some antibodies. This recognized epitope could be disrupted by fixation with formaldehyde. In addition, the antibody neutralization activity was not strictly correlated with its binding affinity.

Reviewer #2: Foot-and-mouth disease virus (FMDV) is a member of the Picornaviridae family that causes severe disease and death in cloven-hoofed animals, including domestic cattle, pigs, sheep, and goats. There are seven FMDV serotypes which do not induce cross-protection in infected animals, and there is need for cross-protective vaccines. This group and others have laid some foundation for this already when they discovered antibodies that are cross-reactive and neutralize Serotype O and A FMDV in previous publications. However, these antibodies were derived from a bovine and camelid source, which have uncharacteristically long heavy chain CDR3 loops or single chain antibodies, respectively. It remained to be seen whether antibodies with more traditional, flatter binding surfaces can also cross-react with multiple FMDV serotypes.

Here, the authors develop a method for discovering monoclonal antibodies that cross-react to multiple FMDV serotypes. The developed method sorts single B cells from FMDV vaccinated pigs using whole virus particles to stain B cells, then uses single-cell RNA-sequencing to determine the sequence of the most abundant FMDV-specific B cells. Through this process, the authors sorted thousands of FMDV-reactive B cells, of which 216 bound to both serotype O and A viruses, and they discovered 10 monoclonal antibodies that neutralize all tested strains of FMDV serotypes O, A (except A/AF72), and Asia1. While most of the B cells from which they derive these antibodies were terminally differentiated, one (pOA-2) was not and its progeny lost the ability to neutralize some strains following additional somatic hypermutation. The authors determined that 9/10 of these cross-neutralizing antibodies recognized a highly conserved RGDL motif in FMDV VP1 G-H loop, which is known to be important for binding the virus’ integrin receptor. The final cross-neutralizing antibodies (again, pOA-2) bridges protomers and pentamers in its interactions with VP2 and VP3. Thermofluor assays indicate that this inter-pentamer binding antibody, but not the RGDL motif-binding antibodies, can cause pre-mature release of the viral genome at physiological temperature.

Response:

Yes, thanks for the comments.

Reviewer #3: In their manuscript, “Discovery, evolution, and recognized antigenic structures of cross serotype broadly neutralizing antibodies from porcine B-cell repertoires against foot-and-mouth disease virus,” Li et al. describe work characterizing the antibody response to FMDV vaccination. The authors immunize a single pig primed with inactivated FMDV (O serotype) and boosted with a bivalent vaccine (A and O serotypes), sort B cells 7 days post-boost for FMDV reactivity and perform B cell receptor repertoire profiling via single B cell sequencing with the 10x Genomics platform on the sorted B cells. The authors devise a clever strategy for isolating cross-serotype antibodies by using the singe B cell sequencing platform to identify shared clones between two independent B cells sorts, on each of the O and A serotypes. The authors then go on to characterize several of these monoclonal antibodies for binding, neutralization capacity, immunogenetics and, for one antibody POA-2, structure. All broad neutralizing antibodies except POA-2 bind to a linear epitope in the G-H loop of VP1 with antibody recognition focused on a RGDL motif that the authors show is conserved across FMDV serotypes owing to its role in receptor binding. The POA-2 structure revealed that it bound to the VP2/VP3 interface another area of cross-serotype conservation. Overall, the manuscript represents an impressive amount of work characterizing the porcine antibody response to FMDV vaccination, however the impact of their findings is tempered by limitations in how the immunogenetic analysis was performed and the manuscript is not helped by the authors’ unpolished writing and general sloppiness in the presentation of the data.

Response:

Yes, thanks very much for the advice. We adjusted data presentation and seriously polished the writing.

Part II – Major Issues: Key Experiments Required for Acceptance
Please use this section to detail the key new experiments or modifications of existing experiments that should be absolutely required to validate study conclusions.

Generally, there should be no more than 3 such required experiments or major modifications for a "Major Revision" recommendation. If more than 3 experiments are necessary to validate the study conclusions, then you are encouraged to recommend "Reject".

Reviewer #1: N/A

Reviewer #2: (No Response)

Reviewer #3:

Question 1

On lines 199-201, the authors state, “…we grouped BCR clones with identical V gene usage and heavy chain CDR3 length into the same lineage”. Grouping B cells into clones based solely on identical V gene usage and heavy chain CDR3 length is inappropriate. Membership in a B cell clone is defined as shared evolutionary descent from a common ancestor with a unique V(D)J rearrangement. V gene usage and heavy chain CDR3 length alone does not define a unique V(D)J rearrangement. There are immunogenetic annotation programs like Partis or Immcantation (which the authors use here for SHM analysis and thus should be familiar with) that can do clonal grouping properly which utilize the CDRH3 sequence to better establish common ancestry. The authors must use those (or alternative programs) to group clones appropriately and reconstruct the lineages for clones with more firmly established clonal relatedness between members.
Response:

Yes, thanks for the advice. We reconstructed the clones and the lineages for clones using the immunogenetic annotation program Immcantation. The 216 common BCR clonotypes constituted 120 unique clonal lineages. These bnAbs were derived from five clonal lineages out of the total 120 clonal lineages. The detailed data were presented in the results and supplemental Table S9.

Question 2
The authors should include a separate table of the immunogenetics of the POA antibodies which establishes the V,D, J gene usage in the heavy and light chains, along with CDR3 lengths and SHM of light and heavy chains. While some of this information is scattered about in Table 1 and S2, it could be presented more clearly and fully in its own table. If the authors had done that, they may have recognized that 8 out of their 9 linear epitope cross-serotype neutralizing antibodies utilize the porcine VK2-10 light chain which seems like an important finding here given the convergence in recognition by those antibodies for the VP1 G-H loop epitope.
Response:

According to the output of the program Immcantation, we listed the clonal group (clone_id), the V,D, J gene usage, SHM, CDR3 lengths of light and heavy chains in the supplemental Table S9 as well as Figure 7. Yes, we added description that “The notable observation is that out of the 9 cross-serotype bnAbs targeting the G-H loop, a total of eight antibodies employed the light chain gene IGKV2-10, indicating a convergence in their recognition of the VP1 G-H loop epitope”.

Question 3
A major limitation in any mutation analysis of antibodies from an animal species with under-sampled and under-sequenced germline Ig gene segments is that allelic variation in Ig genes between individual animals within the same species can result in overestimation of SHM levels. What appears as a mutation is actually a difference between the sequenced pig’s alleles and the germline Ig gene segments in the library used. The mutation frequencies reported for these BCR repertoires and monocolonal antibodies seem quite high for a 9-month old pig. This could be due to under-sampled germline Ig gene segments in pig. The authors should discuss this as a study limitation.
Response:

Yes, we agree with your comments. We stated the study limitation in the discussion section as follows.

“There are several limitations in the immunogenetic analysis of porcine BCR repertoires. Firstly, due to under-sampled and under-sequenced germline Ig gene segments in the utilized reference library, allelic variation in Ig genes between individual pigs within the same species can lead to an overestimation of SHM levels. Some abnormally high SHMs of porcine B cells observed in this study could be attributed to the under-sampling of germline Ig gene segments in pig. Secondly, during the classification of clonal lineages and construction of evolutionary trees using the Immcantation program, certain BCRs failed to obtain their corresponding clonal group or evolutionary tree, such as bnAbs pOA-19 and pOA-19. This phenomenon may arise from incomplete representation of porcine germline Ig gene segments in the reference library used. Lastly, it is important to note that data on antibody repertoires were derived solely from one pig immunized with FMDV antigens. However, whether this response is unconventional or is indicative of a typical response in pigs will not be known until multiple pig repertoires are characterized.”

Question 4
I also found the repertoire-level SHM analysis to lack the appropriate measures and statistical tests given how SHM data is distributed. SHM is not normally-distributed, as is very evident from the violin plots included in Figure 1. However, the authors reported means instead of medians, and applied the Games-Howell statistical test, which assumes normality, when they should have used the Mann-Whitney test. The authors interpretation that in panel 1F plasmablasts are higher in SHM than memory B cells may not hold if they use the appropriate comparison of the medians. Furthermore, is the difference between 0.08 and 0.09 really even biologically meaningful, especially in light of the limitations based on pig allelic variation discussed above? Rather, it would seem the more relevant finding is that the memory B cells mutation frequencies cover a wider range than plasmablasts, and include some unmutated B cells as well as B cells with extremely high mutation frequencies (>20%) whereas the plasmablasts have no unmutated B cells and also no B cells with >20% mutation frequency.

Response:

Yes, thanks for the advice. We reanalyzed the SHM data using non-parametric Mann-Whitney test and showed the medians in violin plot. For comparison of the medians, the SHM median of the plasmablasts (0.09) was also significantly higher than that of memory B cells (0.07). We added the statement of SHM distribution features as per your comments.

To compare the differences in SHMs among different isotypes within each BCR repertoire, we employed the Kruskal-Wallis chi-square test. Given to the limitations based on pig allelic variation, we moved parts of SHM data to supplemental files.

Question 5
The authors conclude from Figure 1G that based on differences of SHM between the O and A-specific populations, that “high affinity IgG antibodies against O/18074 strain may be biasedly produced in the vaccinated pig,” but isn’t the more likely explanation that the A response has simply not had time to mature given the sequencing was performed on samples from only 7 days after the pig was immunized with A (by bivalent boost) whereas the pig had 164 days of maturation time from the original priming immunization with O only?
Response:

Yes, we agree with your explanation. So, we deleted the conclusion “high affinity IgG antibodies against O/18074 strain may be biasedly produced in the vaccinated pig”.

Question 6
The entire results sub-section “Somatic hypermutations balance the breadth and potency of neutralization” misuses the term “clones” and should use the term “members” instead throughout when describing antibodies that are related by evolutionary descent from an unmutated common ancestor (UCA). Again, the conclusions in Figure 3 are tempered by the way the clonal grouping was performed and must be validated by a program such as Partis or Immcantation.
Response:

Yes, using the program Immcantation, we reconstructed the evolutionary tree of pOA2-contianing clonal lineage (clone 66). The results showed pOA2 was also derived from an intermediate stage B cell, and new members 163 and 164 were absorbed in the clonal lineage. So, we expressed the mAb 163 and checked the neutralization activity against all the representatives of the three serotypes. The VNT showed the mAb 163 only neutralized the serotype O strains. The results above were shown in Figure 8, and consistently supported the conclusion “Somatic hypermutations balance the breadth and potency of neutralization”.

Question 7
I found the ordering of the data presentation to be odd. It would seem to be more logical to show the epitope mapping in Fig 6 establishing that 9 of the 10 shared clones map to linear epitopes, and that only pOA-2 binds to a conformational epitope, prior to showing the pOA-2 cryo-EM structures in Figures 4 and 5. As another example, the vaccine regimen is not shown until Figure 2D. It would be better if this was shown as the first panel in Figure 1 and the vaccine regimen described (including the prime and boost and interval) at the beginning of the Results section to orient the reader at the outset..
Response:

Yes, thanks for the advice. We adjusted the order in the revised manuscript.

Question 8
The manuscript would be strengthened by careful editing of the writing. There were various places where the wrong word was used and contributed to the overall impression of a lack of attention to detail by the authors in the preparation of the manuscript. For example, the second word in the abstract is “excavation”, but surely the authors mean “isolated” since they did not extract the antibodies from the dirt. Line 285: “Promoter” should be “Protomer”. Line 977: “Recused” should be “Rescued”.

Response:

Yes, thanks for the correction. We seriously edited the writing.

Part III – Minor Issues: Editorial and Data Presentation Modifications
Please use this section for editorial suggestions as well as relatively minor modifications of existing data that would enhance clarity.

Reviewer #1: Polite request to the authors: Any suggestions, questions, or requests I make are intended to improve the manuscript, ultimately for the benefit of those reading the final published paper. If I suggest that additional information might be useful, my intention is for the extra information to be incorporated into the manuscript (not just provided to me as a reviewer in the rebuttal). Therefore wherever possible, please respond by adding information to the manuscript.
Abstract

Question 9
The abstract is poorly written and confusing and currently does not do justice to the work of this study
line 23 “excavation” wrong word
line 23-25 poor language linking components of the sentence
The description of serotype specific responses/clonotypes/repertoires/antigens is confusing.
Line 31 could be interpreted to mean “all” antibodies targeted RGD, which conflicts with line 36 where the inter-pentamer epitope is introduced.
Line 37 “conservative determinant” what does this mean?
Response：

Thanks for the advice. We corrected these words and rearranged the abstract.
Author summary

Question 10
Also some poor writing, please check/improve if possible.
I would advise against describing your own work as elegant. This is best left for commentary by others.
Response：

Thanks for the advice. We modified the author summary.
Introduction

Question 11
61 Topotypes are not explained.

Response：

Topotype was defined as genetically and geographically distinct evolutionary lineage. For clarity, we modified the sentence as follows: “According to the nucleotide divergence threshold of 15%-20% in the VP1 gene and the epidemic region, there are eleven genetically and geographically distinct evolutionary lineages (topotypes) in serotype O”.

Question 12
63 Suggest “…Asia1 is endemic in parts of Asia…”

Response：Yes, thanks for the suggestion.

Question 13
67 “has been around” rather informal scientific language

Response：Yes, “has been around” was instead of “has persisted”.

Question 14
69-82 make clear you are describing a general situation here (not just FMDV)

Response：Yes, we described a general situation. “In the face of” was instead of “In the context of”.

Question 15
69-70 Suggest “immunologists endeavor to identify and characterize broadly neutralizing antibodies (bnAbs)”.

Response：Yes, thanks for the suggestion. We changed it following your advice.

Question 16
71 “within the body” ?

Response：“within the body” was instead of “in the body”.

Question 17
76 “recognition structures” ?

Response：“recognition structures” was instead of “recognized structures”.

Question 18

77 why is the reference to antiviral drugs relevant for this study?

Response：”antiviral drugs” were deleted.

Question 19
79-82 not really sure what this is supposed to mean

Response：

We mean that the highly conserved structure of the virus antigen is necessary for the survival of the virus under evolution and this make it a potential target for bnAbs against multiple variations. For clear, “species specificity by virtue of its core components' inherent characteristics as well as” was deleted.

Question 20
84 “big” suggest ‘large’

Response：Yes, we changed it following your advice.

Question 21
84-87 not clear. please make clear what was the key finding from previous studies (by you and others) in the area of anti-FMDV B cell repertoires and broadly neutralising antibodies in large animals.

Response：

These key findings from previous studies suggest that the identified bnAbs are peculiar and predominantly recognize antigens using a single structural domain. We added the description.

Question 22
88 How can cattle antibodies be single chain molecules?
Response：

We mean some of cattle antibodies (about 10%-15%) have an ultralong heavy chain complementarity-determining region 3 (HCDR3) that can independently recognize viral antigen without the help of light chain. For clarity, we corrected the sentence as follows: “These reports suggest that bnAbs are peculiar and predominantly recognize antigens using a single structural domain.”

Question 23
93 elegant (see previous comment)

Response：Yes, we deleted.

Question 24
93 mining (wrong word)

Response：

We corrected the sentence as follows: “, we devised an approach for the precise extraction of bnAbs by constructing and dissecting of…”

Question 25
100 “recognized antigenic structures” words in wrong order
Response：

We corrected the sentence as follows: “,the recognized FMDV antigenic structures by bnAbs…”.
Results

Question 26
119-120 confusing. .. if PCR amplification is required before sequencing it should be described in that order.

Response:

Yes, PCR amplification of porcine BCR genes using custom primers was performed before sequencing. We corrected the sentence.

Question 27
121-122 I don’t understand this sentence

Response:

We modified the sentence as follows: “Finally, we successfully established porcine BCR repertoires specific to foot-and-mouth disease virus (FMDV) serotypes O and A, respectively.”.

Question 28
126 suggest “87% of total isotypes (Fig 1B).”

Response: Yes, thanks. We corrected it.

Question 29
131-133 why?

Response:

The definition of porcine clonotype is based on references to other studies like murine or Human B cells for production of antibody.

Question 30
137 indicated the potential presence of conserved antigen structures… ??

Response:

We modified the sentence. “indicated the existence of conserved antigen structures”.

Question 31
138-139 not explained well… is any of this useful: ‘The top highest frequency clonotypes in both O and A repertoires also contained four of the clonotypes shared between O and A.’
Is this finding expected? I was surprised. If broadly reactive responses are rare, why are the shared clonotypes found at the top of the high frequency list? Is there some sort of comment that could be added to address this?
Response:

Thanks for your suggestion. We added the comment following, “This observation appears to contradict the infrequency of broadly reactive responses, which could potentially be attributed to the sequential immunization program employed in this study.”

Question 32
144-146 and Fig 1F I’m not expert on SHM. Can you explain somewhere in the results or figure legend the principle of how the SHM frequency values and Umean (strange symbol) are calculated. I’m guessing the SHM frequency is expressed as a something per something (perhaps this is in the methods). It would be useful to explain why the data in Fig 1F is considered to be ‘notably’ and ‘very significantly’ different, for example the plasmablast population appears to have a bi-modal distribution, with a sub-population of cells with higher SHM, is this what makes it so special? (for example the difference in SHM frequency between serotypes is even greater in Fig1G but this is not highlighted?).
Response:

The SHM indicates the frequency of replacement and silent mutations in the variable region of each heavy chain. We added explain in the figure legend. The Umean indicates the mean of SHM frequency of the total BCRs corresponding to each population. The bi-modal distribution of plasmablast population could result from the sum of SHM frequency of serotype O and A. For further clarification, we reanalyzed the data using the Mann-Whitney test and made the appropriate comparison of the medians. We analyzed the two populations (memory B vs plasmablast) of the serotype O and A-specific repertoires, respectively. The results were listed as supplemental files and supported the SHM frequency of plasmablast population was significantly higher than that of memory B cells in each of the two repertoires.

Considering that the disparity in SHM frequency between serotype O and A could potentially be attributed to variations in immunization timing in pig, we have relocated all findings pertaining to SHM frequency among isotypes and serotypes to supplementary files. Further investigation into the association between SHM and antigenicity of each serotype necessitates more comprehensive data for future studies.

Question 33
Fig1G IgE is there enough data to be confident in these?
Response:

The total numbers of B cells expressing IgE were only 24, accounting for 0.21% of the BCR repertoires. The result may not be confident and therefore we delete the result.

Question 34
149-151 poor sentence

Response:

The sentence has been modified as follows: "Antibody class switch recombination (CSR) was also associated with the maturation of natural antibodies and improvement in SHM."

Question 35
152 significantly increased relative to what? why does the text refer to a % value, this is not shown anywhere in the figure?
Response:

The 10%-11% value was transformed from 0.10-0.11 (the SHM frequency of isotypes IgG and IgA BCR in the O/18074-specific repertoire). For clarity, we modified the sentence as follows: “In O/18074-specific repertoire, the SHM of class-switched isotypes IgG and IgA exhibited a significant increase to 0.10~0.11 compared to that of the IgD isotype.”.

Question 36
155-157 “…difference in humoral response…” what is this difference, can it be explained? “…high affinity ab against O may be biasedly produced…” why? what is the rationale for this statement. Is this referring to data which has not yet been explained?
Response:

Considering that the pig was immunized three times with serotype O strain and only once with serotype A, it is important to acknowledge that the conclusion drawn from the analysis of SHM in the two B cell repertoires may not be robust. Therefore, we have excluded any potential bias in antibody response towards serotype O. We changed the description as follows: “The disparity in SHM between the two repertoires indirectly reflects variations in humoral response to serotype O and A strains in the vaccinated pig, thereby providing information for isolating bnAbs against FMDV.”.

Question 37
162 “the top 10 clones with SHM and the top 10 high frequency clones” why were these types of clones selected, why were these considered to be the most likely to be bNabs out of the 216 clonotypes? e.g. link to the previous findings.
Response:

We primarily select candidates based on the previous study, which screens potential neutralization antibodies against SARS-CoV-2（Cao et al, Cell,2020）. The relatively high somatic hypermutation (SHM) levels may indicate sufficient affinity maturation for the development of potential neutralizing antibodies. The dominant clonotypes with the highest frequency of B-cell receptor (BCR) clones suggest immunodominance towards recognized antigenic epitopes.

Question 38
164 “biological activity” would a more specific phrase be more useful?

Response: The “biological activity” was instead with “reactivity”.

Question 39
167 “binding affinity” where is the data on binding affinity?

Response:

The results of IFA and ELISA mainly reflected the binding capacities of mAbs with FMDV antigen. We corrected the description.

Question 40
168 “only minor variations…” this statement does not match the data in FigS5, some mabs clearly have less activity than others.
why is the data for O so different between IF and ELISA?

Response:

We modified the statement to be “with some variations in their binding capacities as assessed by IFA and/or ELISA.”.

Yes, the results between IFA and ELISA for some mAbs were not consistent. The fixation of infected cells using paraformaldehyde in the process of IFA may disrupt the antigenic structure and accordingly faint fluorescence signals were observed.

Question 41
171-172 “can effectively reflect FMDV-specific humoral immune response” what does this mean?

Response: The statement was not strictly, so we delete it.

Question 42

Why is the IF and ELISA data described in the text as a primary part of the results but the data only shown as supplementary figures?
Response:

The main aim in this study was to reveal and character the broad neutralizing antibodies. IF and ELISA data support the specific of the obtained BCR repertoires.

Question 43
175-177 Virus strains include some not part of the immunisation strategy, how were these chosen?
Response:

We selected representative strains from each topotype that have ever been documented in China.

Question 44
Table 1 I do not find this a good way to summarise the VNT data. The colour coding is not intuitive and is not even explained with a colour key. I would recommend to find a better way to present this data.
Response:

Thanks for the advice. We displayed the data in plot and make the data intuitive using graduated color background.

Question 45
How does the VNT data correlate with the IF and ELISA data?
Response: The correlation between the VNT data and the IFA and ELISA data was not observed during our comparative analysis.

Question 46
191-194 I don’t understand this sentence.
Response:

Yes, we modified the sentence as follows: “However, these bnAbs exhibited comparable average virus neutralization (VN) titers against FMDV serotypes O, A, and Asia1, thereby offering valuable tools for investigating conserved antigenic structures across different serotypes.”.

Question 47

202-203 “ten bnAbs were involved in six clonal lineages…” what does this mean? is involved the best word to use here?
Response:

We reconstructed the clonal lineages of the 216 common BCR clonotypes using immunogenetic annotation program Immcantation. We corrected the sentence as follows: “these bnAbs were derived from five clonal lineages out of the total 120 clonal lineages, ...”.

Question 48
206-209 states “majority of these bnAbs…” but Fig2 only shows 4 antibodies out of 10 (not a majority).
Response:

Yes. We corrected the sentence as follows “According to the evolutionary trees of the bnAbs lineages (Clone_105, Clone_76 and Clone_54), all seven bnAbs targeting the VP1 G-H loop are derived from terminally differentiated B cells that undergo multiple amplifications and accumulation a significant number of SHMs to acquire broad neutralization capabilities.”

Question 49
211 “significantly impacted the breadth of cross-serotype neutralization against FMDV” the evidence for this is not clear from Fig 2C. Is the next sentence intended to provide the evidence for this? If so I don’t understand.
Response:

The data was not confident for the result. So, we delete the statement.

Question 50
214 “evolutionary character” what does this mean?

Response: We delete the sentence.

Question 51
214-222 Why is this not shown earlier? Showing this earlier in the paper would make everything much easier to understand…
Response: Thanks for the advice. We moved the section into the first result.

Question 52
223-233 Apologies I became confused here. Why was bnAb pOA-20 chosen to be used here? There is mention of pigs with repeat immunisation with A antigen but this did not happen in this study? The conclusion of this section needs further explanation.
Response:

We intended to illustrate the cross-serotype antibody response through competing ELISA basis on pOA-20 (cross-neutralization with Asia1) by surveying the field serums from pigs vaccinated with FMDV vaccines. This section may confuse the main idea of this study. So, we deleted this part in the revised manuscript.

Question 53
266-267 The location on the virus capsid of a novel virus-antibody interaction which is broadly neutralising via an unusual destabilising mechanism. This sounds like essential information for this study, I don’t understand why it is relegated to supplementary information!
Response:

Thanks for the advice. We moved this supplementary information into text.

Question 54
272 “promoters” protomers

Response: Yes, thanks for the correction.

Question 55
286-287 sentence doesn’t make sense
Response: Yes, we deleted the sentence.

Question 56
341 “concretely” not the best way to start a sentence
341-345 This confused me, where is this shown?

Response:

We intended to explain which residues are most critical for antigen binding. So, we modified the statement as follows: “Particularly, single substitution occurring at position 146 and 147 completely abolished the binding activity of all these bnAbs.”.

Question 57
346-347 the RGD motif is expected to be highly conserved

Response:

Yes, the RGD motif is highly conservative among seven serotypes FMDV, and represents critical residues for the recognition of FMDV integrin receptor.

Question 58
350 “protrude” protruding

Response: Yes, thanks for the correction.

Question 59
361-363 consider adding amino acid abbreviations for clarity e.g. 143N, 145R, 147D.
Response: Yes, thanks for the advice.

Question 60
385-387 could remind here how these are different

Response:

Yes. We added the statement as follows: “The pOA-1 and pOA-13 recognized the linear VP1 G-H loop with distinct functional antigen determinations at position RGD+2 and +1 respectively, whereas the pOA-2 targeted a conformational epitope at the viral three-fold axis.”.

Question 61
Fif 7A and B identical panels, it would be useful to indicate on the figure which panel is which serotype

Response: Yes, we added the notes.

Question 62
390-394 This seems important and could be explained more carefully . One ab has very striking slow dissociation, is this within normal expected range?
392-394 If this is referring to previous data in this study please make this clear.
Response:

That is a good question. The dissociation rate of virus-antibody complex seems to correlate with efficacy of neutralization (Lantto et al,2002; Tsuji et al, 2022). One possible reason for strike slow dissociation is that “Considering the conservation of RGDL motif, which is bound by pOA-1, on the linear G-H loop epitopes of both strains, the slower dissociation rate observed for O/Tibet/99 compared to A/WH/CHA/09 may also indicate a disparity in the conformation of the flexible G-H loop on viral particles between serotype O and A.”

Question 63
Virus attachment assays: please make clear this is a direct assay of virus attachment at cell surface. Fig S11A does not look like surface labelling and is weak data.
Response:

To enhance quantitation of the virus, our experimental method allowed some amount of viral replication after the adsorption of virus. So we added the statement in the corresponding method and figure legends as follows, “After washing to remove unbound virus, the cells were further cultured for a duration of four hours.”

Question 64
Fig S13 what is this IF supposed be showing, virus at surface or new virus after replication? Legend says entry has been allowed at 37C then mabs added??

Response:

Sorry, we make an error during writing process. The pre-absorption was performed at 4°C for 1h. We corrected this. After incubation with different amounts of mAbs at 37℃ for 1h, the cells were washed with cold PBS to remove unbound virus, then the cells were further cultured for a duration of four hours. This protocol allowed some amount of viral replication in cells and accordingly enhance quantitation of the virus. So, the impressive intracellular fluorescence was observed by IF assay.

Reviewer #2: Overall, the data presented in this manuscript are convincing and support the claim that the authors have discovered several porcine monoclonal antibodies that neutralize multiple FMDV serotypes via distinct mechanisms. The various lines of biochemical evidence additionally highlight the importance of specific residues in the mAb binding sites that are critical for cross-neutralization. However, there is much description of somatic hypermutation (SHM) frequency in Fig 1 that could be moved to supplemental data, as the figures are quite dense overall and these data seem to only describe the intuitive phenomena of having more SHM in response to multiple exposures to a particular antigen (O strain FMDV) and less SHM in response to fewer exposures to a particular antigen (A strain FMDV). While no additional experiments are required for publication, there are some comments for improvement and clarification.
Response：Thanks for the advice. We moved part of the SHM data to the supplemental files.
Big picture:

Question 65
1. The authors frequently refer to the discovered mAbs that neutralize strains from multiple serotypes as “broadly” neutralizing antibodies. Given that none of the discovered antibodies neutralizes all strains that were tested (including one that was included in the inoculation series), and that there were four FMDV serotypes that were not tested, it seems more appropriate to refer to these antibodies as “cross”-neutralizing.
Response：

Thanks for the advice. We selected FMDV representative strains in the three serotypes (O、A and Asia1) that accounted for more than 90% of epidemic worldwide. Additionally, the RGDL motif recognized by these porcine mAbs are conserved among the other four serotypes. We mainly emphasized the bnAbs with cross-serotype neutralization capacity in this study.

Question 66
2. The vaccination strategy and usage of pig(s) is difficult to understand. As best I can tell, all mAbs were isolated from a single pig as detailed in Fig 1. However, the data in Fig 2E may be from a single pig or multiple pigs, and Fig 2F-H must be from multiple pigs, right? What might help is putting the vaccine strategy for the monoclonal antibody source pig (which I understand to be similar to or identical to Fig 2D) as the very first figure of the manuscript. Then if this does indeed differ for the later panels of Fig 2, a separate schematic can be shown there. Similarly, pay very close attention to the use of the singular “pig” and plural “pigs” throughout the text.
Response:

Yes, thanks for the advice. The immunization schematic and antibody response of the pig in Fig 2D-E was moved to Fig 1A-B. In which the pig was used to construct the BCR repertoire and produce mAbs. For Fig 2F-H, via compete ELISA, comparing with the serum samples of the multiple pigs that respectively vaccinated with O, A or the bivalent O and A vaccine, aimed to survey when the cross-serotype antibody response to Asia1 start to produce. To make the paper easier to understand, we deleted the result of Fig 2F-H in the revised version. We also corrected the use of “pig” and “pigs”.

Question 67
3. There are no details of the statistical tests used except for in Fig 1. The rigor of the analysis is therefore hard to evaluate. This needs to be specified for all usages of statistical analysis.
Response: Yes. The data differences between conditions were assessed using unpaired T-test (Holm-Sidak method, α = 0.05) in GraphPad Prism 7.2. We added the statistical tests in Figures legends.

Specific text:

Question 68
The methods for the competitive ELISAs in Fig 2F-H are not described and need to be included.
Response:

The data in this section have little to do with the main idea of this article. The dynamic of cross-serotype antibody response is complex and needs to be further illustrated in detail study in future. So, we deleted the result of Fig 2F-H in the revised version.

Question 69
Fig. S9 shows plaques from rescued escape mutants. While this is good evidence that these viruses do still replicate, it does not tell anything about the extent to which their replication is inhibited (other than differences in plaque size). Data on escape mutant replication kinetics, if already acquired, would be a nice addition and would speak to the ability of the virus to naturally evade cross-neutralizing antibodies.
Response:

That is a good advice. However, we did not collect the data of escape mutant replication kinetics.

Question 70
Line 74: the authors state “Moreover, within the immune milieu, antibodies not only neutralize the pathogens but also trigger their mutations . . .”. This should be phrased differently. Antibodies do not trigger mutations, they select for viruses with mutations that enable escape from antibody recognition.
Response:

Yes, thanks for the correction. We made the statement as follows: “Moreover, within the immune milieu, antibodies not only neutralize the pathogens but also select for viruses with mutations that enable escape from antibody recognition, …”.

Question 71
Line 155: “These information in SHM of the two repertoires indirectly reflected the difference in humoral response to the FMDV strains, suggesting the high affinity IgG antibodies against O/18074 strain may be biasedly produced in the vaccinated pig.” I think the authors are suggesting that there is a qualitative difference in how the vaccinated pig responded to the O strain, but really they just vaccinated three times with O antigens and only one time with A antigen. In other words, this is not a quality of the pig’s B cells, that they respond better to O antigens than A antigens. This is simply a byproduct of the chosen vaccine regimen. Of course the response to an antigen seen many times should be better than to an antigen seen fewer times.
Response:

Yes, the factors that potentially affected level of SHM of the two repertoires are variables, such as the age of pig, immunization times and quality of antigens, and the time of constructing BCR repertoires. The conclusion drawn from the single pig was not convincing. So, we straightforward narrated the data of SHM of different repertoires in the revised version, and part of the results were moved to supplemental files. The conclusion was modified as follows: “The disparity in SHM between the two repertoires indirectly reflects variations in humoral response to serotype O and A strains in the vaccinated pig, thereby providing information for isolating bnAbs against FMDV.”.

Question 72
Line 448: the authors state “Conversely, the evolution of bnAbs targeting a different antigenic structure at the three-fold axis of viral capsids may unveil a distinct role of SHM in enhancing antibody neutralization breadth. The acquisition of an optimal range of SHM is crucial for improving neutralization breadth.” The data presented here surrounding the relationship between SHM and FMDV neutralization breadth are ambiguous. The RGDL-motif cross-neutralizing mAbs are terminally differentiated, but additional SHM reduced neutralization breadth for the pOA-2 lineage. Additionally, in Table 1, multiple mAbs with SHM frequency similar to that for the cross-neutralizing antibodies did not neutralize any of the strains tested. It is certainly true that an optimal range of SHM is important for neutralization, but to correlate this with recognition of a given epitope seems unfounded.
Response:

Yes, for evaluation the relationship between the SHM and neutralization breadth, on the condition that all the members (BCR) were classified into the same clonal lineages and recognized the same epitope. We added the condition of the sentence in discussion.” The acquisition of an optimal range of SHM is crucial for improving neutralization breadth among B cell members within the same clonal lineage and recognizing the identical epitope.” As you mentioned in Table 1, multiple mAbs with SHM frequency similar to that for the cross-neutralizing antibodies did not neutralize any of the strains tested. These mAbs may recognize different epitopes or belong to different clonal lineages.

Question 73
Line 480: the authors state “Presently, anti idiotypic antibodies that target pOA-2 demonstrate significant immunogenicity and can bind with UCA.” Where is this data? If this is published elsewhere, it should be referenced. If it is unpublished data that is not shown, the authors should make this clear.
Response: This is our unpublished data, and modified the sentence.

Question 74
For the pre- and post-attachment mechanisms analyzed in Fig 7 and S13, the authors describe doing all of their analyses immediately within an hour of adsorbing virus to cells. Yet they have impressive fluorescence in their IFA and bands in their wetern blots. This seems more in line with allowing some amount of viral replication after the adsorption of virus. I would like to confirm whether or not the authors allowed some amount of hours or days for the virus to replicate after adsorption to cells.

Response:

Yes. To enhance quantitation of the virus, our experimental method allowed some amount of viral replication after the adsorption of virus. For clarity, we added the description in the method section as follows: “…,the cells were further cultured for a duration of 4 hours to enhance quantitation of the virus, then the final cells were harvested and examined by immunofluorescence…”

Reviewer #3:

Question 75

In Figure 1E, “NA” is reported for two clones’ CDRH3s, but no explanation as to what “NA” means is given in the Figure 1 legend. Surely “NA” is not the amino acid sequence of the CDRH3 for those clones.
Response:

Yes, the presence of “NA” signifies the unavailability of CDRH3 in the B cell due to unsuccessful sequencing of the heavy chain.

Question 76

The two histograms in Figure S4 cannot be distinguished as they are shown. The authors should just show lines without a color fill to help the reader distinguish them. Figure S4 legend is missing a description.
Response: Yes, we corrected the Figure and added a description.

Question 77

Line 493 says “On 5th day after the final vaccination…blood was sampled”. However, Figure 2D seems to suggest blood sampling occurred 7 days after the final vaccination (immunization at day 157 and sampled at day 164). The authors should clarify this inconsistency.
Response:

Thanks for the mention. It is on the 5th day that after the final vaccination. We corrected the data in Figure and method section.

Question 78
The authors should be commended for the level of depth that they went to in order to characterize the antibody repertoire of an FMDV immunized pig. However, whether this response is unconventional or is indicative of a typical response in pigs will not be known until multiple pig repertoires are characterized. The authors should note this as a study limitation.
Response:

Yes, we noted this as a study limitation in the discussion. See it in the revised manuscript.

Question 79
The authors should adequately describe in the text their RGD+1 numbering convention before using it.
Response:

Yes, we added a description. The “first residue after arginine-glycine-aspartic motif (RGD+1)”.

Question 80
Positive and negative control antibodies should be included for the neutralization assay data reported in panels 6I and 6J.
Response:

Yes, thanks for the advice. We added the negative control antibody, clone PLY55, that is a porcine-derived mAb but negative for FMDV. Positive control antibody was not available, because this study firstly reported of porcine cross-serotype bnAbs.

Question 81
Figure 6 legend says “(I,I)” but should be presumably say “(I,J)”.
Response: Yes, thanks for the advice. We corrected it.

Question 82
Figure 7 has two panels labeled “E”, presumably the one on the right should be labeled “F”.
Response: Yes, thanks for the advice. We corrected it.

Question 83
The POA-13 antibody is missing from Table S2.
Response: Yes, we reanalyzed the clonal lineages and pOA-13 was included in the revised Table S9.

Question 84
Line 512: “High-through sequencing” should be changed to “High-throughput sequencing”

Response: Yes, thanks for the advice. We corrected it.
