# Supplementary material for: Discovery, recognized antigenic structures, and evolution of cross-serotype broadly neutralizing antibodies from porcine B-cell repertoires against foot-and-mouth disease virus
Source: PLoS Pathog. 2024 Oct 15;20(10):e1012623. doi: 10.1371/journal.ppat.1012623 (PMC11508087; doi:10.1371/journal.ppat.1012623)
Supplement: Attachment — Submitted filename: Response to conments.docx [file ppat.1012623.s005.docx]

Dear editors,

Thanks very much for checking the revision and advices for improvement manuscript. We made the following modification as per your suggestions.

(1) We agree with the comments from reviewers and rephased the conclusions about the pre- and post-attachment assays. We made the point-to-point responses to reviewers.
(2) The sequences of all the porcine-derived monoclonal antibodies have been deposited in the GenBank (Accession Numbers PQ305442 to PQ305481). This was stated in the **Data availability** section.
(3) The detail methodology on virus production and (for the product used for vaccination) inactivation process was added in **Materials and Methods** section.
(4) We specified unit on the y axis of Fig 1H as “% nt SHM”, and also made corresponding changes in Figs 2, 7, S5. The SHM was calculated based on mutations per nucleotides in the entire variable region sequence (VDJ region) of immunoglobulin heavy chain (VH). The frequency of SHM was showed in percentage (% nt SHM). For very few mAbs, such as pOA-11 and pOA-19, their SHMs were calculated basis on mutations per nucleotides in the V gene segment only, because of the failure in identifying the sequences which clones they belong to. Therefore, for Fig 2, we added the annotation for clarity "$ noted the SHM data of this mAb was discriminatively calculated basis on V gene segment of heavy chain." in the legend. We added the details on calculation of SHM in the **Materials and Methods** section.

Additionally, all the figures had been checked by PACE and the references were also checked. All relevant data are within the paper and its *SI appendix.* The manuscript was meticulously revised and polished by the authors to enhance its clarity and readability, thereby hoping to meet the stringent standards of your professional and academic publication.

Thanks again!

Best wishes,

Kun Li

**Reviewer's Responses to Questions**

**Part I - Summary**
Please use this section to discuss strengths/weaknesses of study, novelty/significance, general execution and scholarship.

Reviewer #1: Resubmission PPATHOGENS-D-24-00480R1 from Li and Wu et al.

Thank you to the authors for the additional work to modify and improve this manuscript.

Response: Thanks very much for the comments.

Reviewer #2: Overall, I am pleased with the modifications made by the authors to the manuscript. They adequately addressed the minor concerns I had with the previous version, and the manuscript is nearly fit for publication. However, I have noticed that the conclusions drawn from the pre/post attachment experiments are not quite in-line with the way the experiments were performed. Rather than changing or doing new experiments, I think this can be addressed with some modification of the text. I have also included some minor textual recommendations that would improve the reading of the manuscript, were they to be incorporated.

Response:

Thanks very much for the advices. These comments are valuable and contributed great for improvement of the manuscript. We adjusted the text in accordance with the advices and seriously refined the writing.

Reviewer #3: The authors' revisions substantially have strengthened the manuscript. I recommend accepting the manuscript for publication.

Response: Thanks very much for the comments.

**Part II – Major Issues: Key Experiments Required for Acceptance**
Please use this section to detail the key new experiments or modifications of existing experiments that should beabsolutely required to validate study conclusions.

Generally, there should be no more than 3 such required experiments or major modifications for a "Major Revision" recommendation. If more than 3 experiments are necessary to validate the study conclusions, then you are encouraged to recommend "Reject".

Reviewer #1: N/A

Reviewer #2: The authors need to include the entire variable region sequences of their mAbs. At the very least they need to include this for the 10 bnAbs that they focus on, but really any mAb mentioned in the manuscript should have the sequence published. This is field standard to allow for reproducibility for other groups. This could be in a separate supplementary Excel table as one possible suggestion.

Response:

Yes, the sequences of all the porcine-derived monoclonal antibodies have been deposited in the GenBank (Accession Numbers PQ305442 to PQ305481).

Reviewer #3: None

**Part III – Minor Issues: Editorial and Data Presentation Modifications**
Please use this section for editorial suggestions as well as relatively minor modifications of existing data that would enhance clarity.

Reviewer #1: N/A

Reviewer #2: Regarding the pre- and post-attachment assays (shown in Figs. 9C-H and S10 and S12), the conclusion the authors draw from the data are not as definitive as they present them. First, for the pre-attachment assay in which the mAbs are mixed with virus prior to attachment to cells, it is certainly possible that the mAbs function by blocking the receptor as the authors conclude. However, it is also entirely possible to opsonize mAbs to a virion, then have that virion bind to an attachment factor or receptor, then have the mAbs block some step of entry or uncoating that comes after the attachment step. Therefore, the way to better interpret these data is that the mAbs are *capable* of blocking attachment to cells. But this is not definitive evidence of blocking attachment as the authors present it. While this may seem subtle, it’s an important distinction.
Response:

Thank you very much. We completely agree with your comments and made the modification as per your suggestion.

Question 1
Second, regarding the post-attachment assays there is a fatal flaw that I now realize with the added detail provided around these methods, which is why I asked for them in the first review. The authors pre-adsorb virus to cells at 4°, but then they add antibody at 37° for an hour. In doing this, they allow for the endocytic machinery to start back up. Therefore, the viruses that adsorbed to the cell have a chance to endocytose before the mAbs have the chance to fully opsonize bound virions. Instead, the antibodies should have been allowed to opsonize the cell-adsorbed virions at 4° (which can be for longer than 1 hr, that’s not a problem). By never including a 37° incubation in the middle of the protocol, it is possible a stronger neutralization effect would have been detected. All of this considered, the correct interpretation of these data is that the small amount of post-attachment inhibition seen by pOA-2 is certainly real, but the way this assay was performed may well have underestimated the post-attachment neutralization this mAb is capable of. And the lack of post-attachment neutralization seen for the other mAbs may simply have been missed due to the way the assay was performed.
Response: Thank you very much. We agree with your explanation. We stated the deficiency of the employed post-attachment assays in the result section as follows.

“However, the observed results could result from the employed post-attachment assay that allowed incubation of antibody with the pre-absorbed cells with viruses at 37°C for 1 h. This protocol inadvertently allowed for the reactivation of endocytic machinery, enabling adsorbed viruses to undergo endocytosis before the mAbs could fully opsonize bound virions. Taking these factors into consideration, it is evident that the observed minimal post-attachment inhibition by pOA-2 is certainly real, but the way this assay was performed may well have underestimated the post-attachment neutralization this mAb is capable of. And the lack of post-attachment neutralization seen for the other mAbs may simply have been missed due to the way the assay was performed.”

Question 2
Nowhere in the Materials and Methods do the authors describe a) how they make their virus preps (specifically what are the cell lines used to make them, what are the purification steps), nor b) how the viruses are inactivated for the vaccines. Please include these details.

Response:

Thanks very much for the advice. We elaborated on the procedures “Virus production, inactivation and purification of 146S antigens” in the Materials and Methods section as follows.

**“Virus production, inactivation and purification of 146S antigens**

FMDV (O/HN/CHA/93, O/18074, O/Tibet/99, A/WH/CHA/09, or A/AF72) was propagated in the baby hamster kidney cells (BHK-21) that have been cultured adherently in a 5-layer cell factory system (NEST, China) at 37℃ with 5% CO_2_ for 48h. After incubation with FMDV under the same conditions for 12 h, the cell supernatant was collected following three freeze-thaw cycles. Viruses in supernatant were inactivated by treating with 1.2% Binary ethylenimine (BEI) at 30℃ for 28 h, followed by the addition of a stop solution containing 4% sodium thiosulfate to neutralize the effect of BEI. The deactivated viruses were checked in BHK-21 cells for three successive passages, and no CPE was observed within each passage for a duration of 48 h, indicating complete viral inactivation. For purification of 146S antigen, approximately 1 liter of the inactivated viruses were precipitated by incubating at 4°C overnight in 8% (w/v) PEG 6,000. The precipitated virus antigens were harvested by centrifugation at 3,500 g for 1 h at 4°C, followed by resuspension in 50 ml PBS (137 mM NaCl, 2.7 mM KCl, 50 mM Na_2_HPO_4_ and 10 mM KH_2_PO_4_, pH=7.6). Subsequently, viral antigens were pelleted through a cushion of 30% (w/v) sucrose in PBS by centrifugation at 35,000×g for 4 h. The sucrose was removed from the pellet, and 500 μl of PBS was added to cover the pellet. The supernatant was further purified over a 20–60% sucrose gradient and fractionated by centrifugation at 35,000 g for 4 h at 4°C.The fractions were analyzed by negative stain electron microscopy, and the fraction corresponding to 146S antigen was transferred to a 100 kDa MWCO centrifugal filter for buffer exchange with PBS to remove the sucrose. The concentrations of the 146S antigens were quantified by a spectrophotometer at 260 nm (where an optical density of 7.6 = 1 mg/ml), and immediately used for subsequent experiments.”

Question 3
The y axis of Fig. 1H and the “SHM” column in Fig. 2 has no units. Frequency of SHM based on what calculation? Mutations per amino acid in a given sequence? If you could explain this metric to the reader a little better, it would help a lot as you reference SHM rate often during the manuscript, including being the basis for the entire paragraph of lines 154-173. It seems clear that larger numbers mean more mutations, but knowing the details of how this is calculated with an extra sentence or two in the methods would be helpful.
Response:

Thanks very much for the advice. We modified the y axis of Fig 1H to “% nt SHM”. The SHM was calculated based on mutations per nucleotides of entire variable region sequence of heavy chain. We elaborated on the calculation of SHM in the Materials and Methods section as follows.

“The SHM was calculated based on mutations per nucleotides in the entire variable region sequence (VDJ region) of immunoglobulin heavy chain (VH). The frequency of SHM was showed in percentage (% nt SHM). For very few mAbs, their SHMs were calculated basis on mutations per nucleotides in the V gene segment only, because of the failure in identifying the sequences which clones they belong to.”

Question 4
Table S9 is missing. I’m guessing this might have the sequences of the mAbs.

Response:

No, the table S9 have provided the full information and the editor has confirmed this.

Question 5
The panels Fig 4C and D would be fine to move to the supplement.

Response: Thanks very much for the advice. We moved Fig 4C and 4D to the supplementary materials section.

Minor text recommendations:

Question 6
Line 69: remove “most” as part of “most noticeable”. No need for a superlative here.

Response: Thanks very much for the advice. We removed the “most”.

Question 7
Line 72: what exactly is meant by “diverse viral variations”? Different viruses? Mutants within specific groups of viruses? The word choice is vague and confusing.

Response: Yes. For clarity, we rephase it as follows. “the genetic diversity of viral variations”.

Question 8
Line 91: instead of “Y-shaped” the use of “bivalent” would be more appropriate.

Response: Yes, thanks very much for the advice. We made the change.

Question 9
Line 102: change wording to “the antigenic structures recognized by bnAbs reveal”

Response: Yes, we made the change.

Question 10
Line 103: change “the most direct” to “a direct”.

Response: Thanks very much for the advice. We made the change.

Question 11
Line 120: “sophisticated” is subjective. Remove this word.

Response: Yes, thanks very much for the advice. We removed the “sophisticated”.

Question 12
Line 149: “suggested” would be more appropriate than the term “indicated”, as the existence of conserved antigenic structure is only one possible interpretation of the data. I agree it’s a reasonable interpretation, but it is not the only one.

Response: Yes, thanks very much for the advice. We made the correction.

Question 13
Line 170: “it all accumulated rather than high SHM in pig induced by two different strains”. This wording is confusing, please clarify.

Response: Thanks very much for the advice. We rephased it as follow “both of repertoires have rather high SHM accumulation up to about 10%.”

Question 14
Line 242: “The flexibility of G-H loop of VP1 on viral surface made the virus-receptor complex conformational heterogeneity”. This wording is confusing, please clarify.

Response: Yes, thanks very much for the advice. We modified the sentence as follows. “The flexibility of G-H loop of VP1 on viral surface made the conformation of virus-receptor complex diversiform”.

Question 15
Line 257: the authors use “drove the variations” when it is likely that they mean “selected for mutations”. Please clarify.

Response: Yes, thanks very much for the advice. We made the change.

Question 16
Line 979: in the description for panel 7A, specify why you use the different colors.

Response: These bnAbs showing with different colors aimed to highlight their difference in the V gene usage of light chain. We added the explanations in the figure legends section as follows.

“Basis on the difference in the V gene segment usage of light chain, 10 bnAbs were colored with blue, light blue, and pink backgrounds, respectively. Specifically, the 8 bnAbs employed the same light chain V gene IGKV2-10*02 on a blue background, while bnAb pOA-13 utilized IGLV8-16*01 on a light blue background. Additionally, bnAb pOA-2 employed IGLV8-19*02 on a pink background.”

Reviewer #3: None
